# Nanozyme-Engineered Hydrogels for Anti-Inflammation and Skin Regeneration

**DOI:** 10.1007/s40820-024-01323-6

**Published:** 2024-02-06

**Authors:** Amal George Kurian, Rajendra K. Singh, Varsha Sagar, Jung-Hwan Lee, Hae-Won Kim

**Affiliations:** 1 Institute of Tissue Regeneration Engineering (ITREN), Dankook University, Cheonan, 31116 Republic of Korea; 2 Department of Nanobiomedical Science & BK21 NBM Global Research Center for Regenerative Medicine, Dankook University, Cheonan, 31116 Republic of Korea; 3 Department of Biomaterials Science, School of Dentistry, Dankook University, Cheonan, 31116 Republic of Korea; 4 UCL Eastman-Korea Dental Medicine Innovation Centre, Dankook University, Cheonan, 31116 Republic of Korea; 5 Cell and Matter Institute, Dankook University, Cheonan, 31116 Republic of Korea; 6 Mechanobiology Dental Medicine Research Center, Dankook University, Cheonan, 31116 Republic of Korea

**Keywords:** Nanozymes, Hydrogels, ROS scavenging, Anti-inflammation, Skin regeneration

## Abstract

Inflammatory skin disorders can cause chronic scarring and functional impairments, posing a significant burden on patients and the healthcare system. Conventional therapies, such as corticosteroids and nonsteroidal anti-inflammatory drugs, are limited in efficacy and associated with adverse effects. Recently, nanozyme (NZ)-based hydrogels have shown great promise in addressing these challenges. NZ-based hydrogels possess unique therapeutic abilities by combining the therapeutic benefits of redox nanomaterials with enzymatic activity and the water-retaining capacity of hydrogels. The multifaceted therapeutic effects of these hydrogels include scavenging reactive oxygen species and other inflammatory mediators modulating immune responses toward a pro-regenerative environment and enhancing regenerative potential by triggering cell migration and differentiation. This review highlights the current state of the art in NZ-engineered hydrogels (NZ@hydrogels) for anti-inflammatory and skin regeneration applications. It also discusses the underlying chemo-mechano-biological mechanisms behind their effectiveness. Additionally, the challenges and future directions in this ground, particularly their clinical translation, are addressed. The insights provided in this review can aid in the design and engineering of novel NZ-based hydrogels, offering new possibilities for targeted and personalized skin-care therapies.

## Highlights

- Nanozyme-based approaches to produce therapeutic hydrogels.
- Enzymatic mechanisms and multifunctional roles of nanozyme-engineered hydrogels for skin therapy.
- Therapeutic actions of nanozyme-engineered hydrogels in inflamed skin tissues.
- Mechanical and immunological aspects of skin therapy guided by nanozyme-engineered hydrogels.
- Promising directions and challenges of nanozyme-inspired hydrogel platforms.

## Graphical abstract

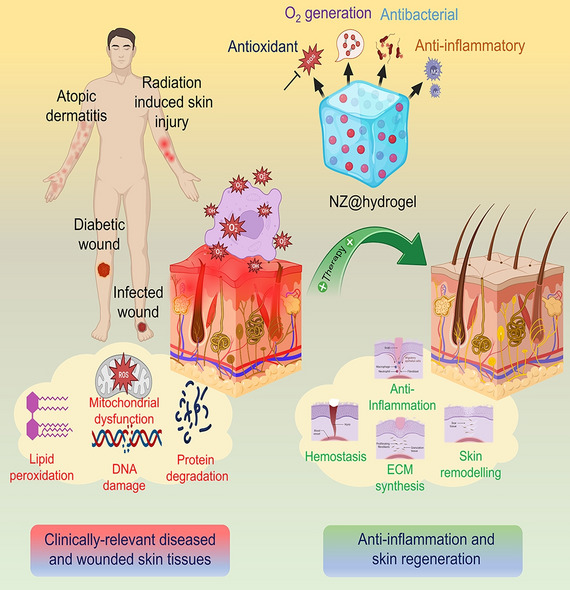

## Introduction

Skin impairment resulting from various factors such as injuries, infections, and diseases has become a pervasive global concern [1]. Under normal circumstances, the skin possesses remarkable regenerative abilities orchestrating a well-coordinated sequence of events involving inflammation, hemostasis, proliferation, and remodeling of the extracellular matrix (ECM) [2]. These phases of skin wound healing depend on communication between cells, bioactive substances, and the ECM. However, chronic conditions such as burns, infection, or dermatitis can disrupt such interactive healing processes, necessitating targeted interventions to promote tissue regeneration [3].

The pathophysiology of skin damage involves a complex interplay of cellular and molecular processes [4]. Initially, hemostasis rapidly halts bleeding and covers the damaged protective barrier by activating blood-clotting mechanisms. This is followed by an inflammatory phase characterized by the release of pro-inflammatory cytokines and chemokines, which recruit immune cells, such as neutrophils and macrophages, to the injury site [5]. Neutrophils, the initial responders, clear bacteria and cellular debris, while macrophages coordinate subsequent wound healing phases [6]. The next stage involves tissue repair and regeneration. Fibroblasts from connective tissue migrate to the injured area, synthesizing collagen, the primary skin structural protein [7]. Collagen provides mechanical strength, gradually replacing the initial blood clot. As the wound heals, new blood vessels form to supply oxygen and nutrients, while keratinocytes, the main epidermal cells, proliferate and migrate to restore the protective skin barrier [8]. Re-epithelialization is crucial for wound closure. In chronic skin conditions such as dermatitis, pathophysiology involves dysregulated immune responses, genetic predispositions, and persistent inflammation cycles [9]. Additionally, external factors such as radiation and infections can also lead to ROS production, causing oxidative stress and skin inflammation [10].

To address these challenging pathological skin conditions, significant attention has been directed toward tissue engineering techniques to regenerate inflamed skin tissues that offer potential alternatives to conventional wound healing approaches [11]. Skin tissue engineering (STE) is a multidisciplinary field that combines principles from biology, materials science, and engineering to create functional and biocompatible skin substitutes for regenerative purposes [12]. The primary goal is to develop artificial or bioengineered skin that can replace or assist damaged or diseased native skin in patients [13]. While traditional healing methods are valuable, they have limitations compared to tissue engineering techniques. Traditional approaches rely on the body’s natural healing process, which can be insufficient for chronic wounds, severe burns, or infected wounds [14]. They may struggle to address challenges effectively like infection control, pain management, and excessive scarring [15]. Moreover, traditional methods often require frequent interventions and prolonged hospital stays, resulting in increased healthcare costs. In contrast, tissue engineering offers a more controlled and expeditious approach to tissue repair, reducing infection risk, minimizing scarring, and providing a platform for personalized, patient-specific solutions [16, 17]. These advantages make tissue engineering a compelling alternative, particularly in cases where traditional methods have proven inadequate or suboptimal for severe and chronic skin wound healing.

Although tissue-engineering approaches hold promise, they also encounter considerable impediments due to the diverse characteristics of skin tissues, such as variations in morphology, biochemical composition, and mechanical properties [18]. The absence of complete replacements that accurately replicate the complex nature of natural skin tissue further underscores these difficulties. Nevertheless, researchers are striving to overcome these hurdles by integrating essential components such as advanced nano-biomaterials to develop new platforms that can eventually generate artificial tissues [19].

The rapid progress in nanotechnology has led to significant advances in the development of nanomaterials known as nanozymes (NZs) which exhibit enzyme-like activities [20, 21]. Unlike natural enzymes which require specific physiological conditions to function as catalysts, NZs maintain their biocatalytic activity even under extreme temperatures and pH levels and are even resistant to degradation [22]. Gao et al. achieved a remarkable breakthrough in the field by unveiling an artificial peroxidase (POD) enzyme based on ferromagnetic magnetite (Fe_3_O_4_) nanoparticles (NPs) [23]. Following this pioneering work, extensive investigations have been carried out on several nanomaterials including metallic, metal oxide-based, and carbon-based nanomaterials to explore their inherent enzymatic activities, particularly the POD, oxidase (OXD), catalase (CAT), and superoxide dismutase (SOD)-like activities [24–26]. Currently, NZs and NZ-based platforms are the subject of extensive investigation due to their potential applications in various fields such as tissue engineering, immunoassays, biosensing, disease diagnosis, and therapy [27–30].

Despite substantial progress in developing various NZs for biomedical purposes, unraveling the fundamental factors that influence their catalytic performance remains a challenge. Moreover, a comprehensive understanding of the catalytic mechanisms of NZs is indispensable for the rational design of new NZs with essential catalytic properties [30, 31]. Recent studies have highlighted the diverse biomedical applications of NZs including chemo-dynamic therapy (CDT), treatment of bacterial infections, and management of diseases associated with reactive oxygen species (ROS) [32]. Consequently, the advances in the field of NZs hold significant potential to transform the treatment of inflamed skin tissues and offer novel insights into the widespread acceptance of these materials.

Over the past few decades, hydrogels have gained significant recognition and are used for STE owing to their intrinsic biocompatibility and precise tunability of physicochemical properties [33–35]. Hydrogels with their tunable properties closely resembling the natural ECM of the skin provide an environment that supports cell growth, proliferation, and tissue regeneration [36, 37]. Additionally, hydrogels can be designed to enable controlled drug release, encapsulate cells for transplantation, and incorporate bioactive molecules to enhance cellular interactions and signaling [35]. These properties make hydrogels a highly appealing platform for advanced therapies for skin regeneration presenting potential solutions for personalized skin-care treatments.

The application of NZs offers a promising direction to address these challenges by effectively mitigating molecular oxidation events in the biological environment and restoring an appropriate balance of ROS in skin tissues [38]. However, their efficacy is often hindered by limited bioavailability and bioactivity when directly applied to wounds. Consequently, there is a compelling need to integrate hydrogel technologies to develop NZ-engineered hydrogels (NZ@hydrogels) with the capacity to precisely regulate ROS levels and thereby target a wide range of wounded and pathological skin conditions [39].

NZs can reinforce the mechanical strength of hydrogels, enabling them to withstand mechanical stresses and mimic the natural properties of skin tissues [40]. Moreover, NZs contribute to the stability of hydrogels by preventing undesired degradation while maintaining a stable microenvironment. For example, Wang et al. reported that NZ incorporation led to improved mechanical properties, increasing the stability of hydrogels [41]. Similarly, Li et al. demonstrated that the integration of NZ into hybrid hydrogels improved the mechanical properties, allowing them to protect surrounding tissues against damage [42]. Similar findings have also been reported in various biomedical applications [43–45].

Notably, NZs promote vascularization ensuring a sufficient supply of nutrients and oxygen (O_2_) to the developing tissue [46]. Besides, they enhance cellular infiltration and improve the integration of hydrogels with the surrounding tissues and also exhibit anti-inflammatory properties that mitigate the potential inflammatory responses induced by hydrogels [47]. Thus by integrating NZs, the effectiveness of hydrogels can be substantially improved resulting in enhanced results for STE [48]. For instance, a recent study by Kim et al. reported the use of nanoceria (nCe) with catalytic properties incorporated within a hydrogel matrix for treating atopic dermatitis (AD) [49]. This study suggests that incorporating nCe into hydrogels holds great promise for enhancing the bioavailability and efficacy of NZs. Another study by Li et al. developed a multifunctional, shape-adaptable hydrogel incorporating tannic acid (TA) bound Fe-decorated molybdenum disulfide nanosheets (MoS_2_@TA/Fe NSs) which exhibited high antibacterial and antioxidant effects [50]. Additionally, Jin et al. explored SOD-mimicking Ni_4_Cu_2_ hollow nanospheres by incorporating them into thermosensitive hydrogels to enhance acute wound healing [51]. Hence, there has been a substantial surge in the development of therapeutic hydrogels with enzymatic properties aimed at pushing forward the frontiers of skin wound therapies.

The multifunctionality of NZ@hydrogels extends well beyond their enzymatic activities [47]. One notable example is the enhancement of hydrogel conductivity. NZs, especially those based on carbon materials, can be effectively combined with hydrogels to introduce electrical conductivity [52–54]. This property is invaluable in regenerative medicine, where it enables the creation of conductive pathways for electrical signaling, facilitating the growth and differentiation of cells. Moreover, certain surface-engineered NZs can contribute to the enhanced adhesiveness of hydrogels, ensuring better integration with biological tissues [55, 56]. This adhesiveness is particularly advantageous in wound dressings and surgical applications, where safe and biocompatible tissue adhesion is critical.

Certain NZs also possess photothermal properties, allowing them to convert near-infrared (NIR) radiations into localized heat [57, 58]. When integrated into hydrogels, these NZs enable on-demand photothermal therapy (PTT). This property has significant implications in antibacterial therapy, where the hydrogel is triggered to release heat upon exposure to NIR light, effectively destroying bacteria, and facilitating rapid healing of infected wounds [59, 60]. The Incorporation of magnetic NZs into hydrogels provides another level of functionality [61]. Magnetic NZs can be directed and controlled using external magnetic fields, allowing for targeted drug delivery for tissue healing or even for enhanced diagnostic imaging [62]. The incorporation of NZs also imparts hydrogels with antimicrobial properties to combat infections and enhance the wound healing process [63, 64]. It is also reported that some NZs facilitate in situ gelation of hydrogel precursors, allowing for minimally invasive delivery and on-demand hydrogel formation within the body, which are particularly advantageous for localized tissue therapy [65]. These multifunctional features make NZ@hydrogels a promising candidate for a wide range of applications, including smart wound dressings and tissue engineering scaffolds.

**Fig. 1:**
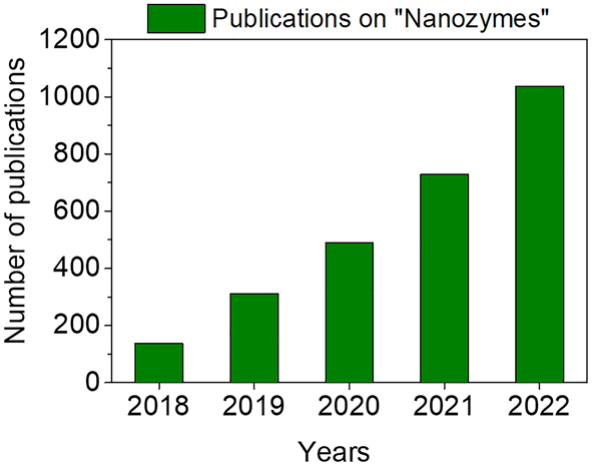
Number of NZ-related articles published in the last 5 years according to Web of science (report acquired using the keyword ‘nanozymes’), indicating the scope and importance of this rapidly emerging platform

Figure 1 illustrates the increasing number of NZ-related articles published in the last 5 years, which highlights the scope and importance of this rapidly emerging platform. In this context, this review aims to emphasize the strides made in nanotherapeutic approaches for the treatment of injured and diseased skin tissues. We discuss the strategies involved in developing new formulations of NZ@hydrogels evaluating their efficacy in promoting skin wound healing across various pathological conditions and further elucidate the underlying chemical, mechanical, and biological mechanisms that contribute to their effects. We also address the challenges faced in this domain and explore emerging prospects, particularly concerning their clinical applications. We believe that the insights provided in this review will aid in the development and design of novel hydrogels based on NZs that offer new possibilities for targeted and personalized skin-care therapies.

## Functional Roles of Hydrogel in NZ@Hydrogel System

Hydrogels play a key role in the functionality of NZ@hydrogels designed for skin regeneration [66]. Hydrogels stabilize NZs, preventing their agglomeration and ensuring uniform distribution throughout the composite structure [44]. For instance, Chen et al. developed a cerium-based metal–organic framework (MOF) hydrogel using an in situ 3D-printing technique that displayed uniform NZ distribution [67]. The in situ synthesis allowed for the direct incorporation of Ce-based MOF NZ into the porous network, enhancing their dispersion and concurrently improving the mechanical properties of the printed hydrogel. The hydrogel-supported composite can closely mimic the natural skin ECM due to its high water content and gel-like property that facilitates cell adhesion, proliferation, and migration, which are essential for tissue regeneration [68]. Hydrogels also serve as a 3D scaffold for immobilizing NZs, providing stability and protection to NZs from harsh environmental conditions such as pH or temperature change [69]. For example, Baretta et al. developed a NZ@hydrogel by incorporating Prussian blue (PB) NZ and glucose oxidase (GO_*x*_) into carboxymethyl cellulose (CMC)-based hydrogel for the colorimetric estimation of glucose [44]. This composite employs CMC for gelation and crystalline PB NZ, functioning as both a cross-linker for the hydrogel network and an NZ with POD-like activity. The CMC-based hydrogel network acted as a biocompatible scaffold for immobilizing GO_*x*_ without compromising its catalytic activity. Additionally, the hydrogel provided a confined environment that facilitated biocatalytic cascade reactions between the enzyme and NZ, and the PB–CMC–GO_*x*_ hydrogel composite exhibited enhanced sensitivity for colorimetric glucose detection. Compared to a combination of separately immobilized GO_*x*_ and PB–CMC, this system demonstrated improved glucose detection and enhanced stability.

Hydrogels also offer controlled release ability, enabling gradual and targeted delivery of NZs, which is particularly advantageous in chronic skin defects [70, 71]. For instance, Li et al. utilized selenide-bound polydopamine-reinforced composite hydrogels (DSeP@PB) with on-demand degradation and light-activated NZ release properties for regenerating diabetic skin wounds [42]. The incorporation of PB NZs resulted in superior injectability and flexible mechanical properties in the resultant NZ@hydrogel. The incorporation of dynamic diselenide introduces the capability for on-demand degradation in response to reducing or oxidizing conditions, as well as light-triggered release of NZs. Interestingly, injectability was assessed through simulation experiments, revealing that the hydrogels were easily injectable, could flow smoothly through a needle, and could recover into stable hydrogels. The DSeP@PB hydrogel exhibited potent antibacterial, ROS-scavenging, and immunomodulatory effects, protecting against oxidative damage and inflammation in cells. Animal studies further demonstrated that the NZ@hydrogel exhibited the most effective wound-healing activity by promoting angiogenesis, collagen deposition, and suppressing inflammation. The synergistic advantages of DSeP@PB, including on-demand degradation, light-triggered release, flexible mechanical robustness, antibacterial properties, ROS-scavenging capabilities, and immunomodulatory effects, position it as a promising hydrogel dressing for safe and effective therapeutic applications in diabetic wound healing.

Sometimes, the hydrogel itself can possess bioactive properties, such as antibacterial or ROS scavenging abilities, which, when combined with NZs, can synergistically enhance the overall therapeutic potential of NZ@hydrogels [72, 73]. For example, Zhao et al. utilized polyvinyl alcohol (PVA) cross-linked by ROS-responsive linker called N^1^-(4-boronobenzyl)–N^3^-(4-boronophenyl)–N^1^, N^1^, N^3^, N^3^-tetramethylpropane-1,3-diaminium (TPA), which serves effectively as ROS scavenger, aiding in wound closure by diminishing ROS levels and fostering the presence of M2 phenotype macrophages in wound microenvironment [74]. This hydrogel was infused with both mupirocin (an antibiotic) and granulocyte–macrophage colony-stimulating factor (GM-CSF), a growth factor recognized for its tissue-regenerating properties. Within the wound microenvironment, the hydrogel displayed the capability to counteract ROS, thereby promoting wound healing. This effect is achieved by regulating pro-inflammatory cytokine levels, increasing M2 polarization of macrophages, and stimulating angiogenesis and collagen synthesis. Simultaneously, the hydrogel, guided by ROS-responsive cleavage of TPA, underwent gradual degradation, leading to a controlled release of mupirocin and GM-CSF, which hinders bacterial infections and accelerates wound repair. This has been validated in vivo by its effectiveness in treating various types of wounds, including challenging-to-treat infected diabetic wounds.

It is also known that the bioavailability of NZs is enhanced within hydrogels due to their ability to facilitate absorption and retention within wound tissues, which ultimately accelerates wound healing [70]. For instance, Chao et al. incorporated a metal–organic framework (MOF) NZ with antioxidant enzyme-like properties into a thermosensitive hydrogel, poly(lactic-co-glycolic acid)-polyethylene glycol-poly(lactic-co-glycolic acid (PLGA-PEG-PLGA), introducing an efficient antioxidative system for the chronic wound healing of diabetic rats [75]. In this context, the hydrogel effectively anchored the MOF NZ at the wound sites, ensuring continuous and sustained treatment. Due to its thermosensitivity, this hydrogel undergoes a transition from a sol to a gel phase, rendering it well-suited for application as in situ wound dressings. The lap shear test revealed that the NZ@hydrogel exhibited a substantial quantitative adhesive strength of up to 2.52 kPa on porcine skin, providing additional confirmation of its satisfactory tissue adhesiveness. Notably, therapeutic hydrogel necessitated only a single application throughout the entire treatment duration, in contrast to human epidermal growth factor Gel (HEGFG), which required daily application. This underscores the therapeutic advantages of MOF NZ with enduring efficacy.

Hydrogel itself often plays a key role in preventing infections during the wound healing process [76]. They create a barrier over the wound site, protecting it from external contaminants such as bacteria and dust. By maintaining a moist environment that is conducive to optimal wound healing, hydrogels can inhibit the growth of harmful microorganisms as many pathogens thrive in dry conditions [71]. Moreover, many hydrogels are known to have intrinsic antibacterial properties that aid the wound healing process [77]. For instance, Yang et al. formulated a composite cryogel through the copolymerization of carboxymethyl chitosan with double bonds (CMCSG) and thermosensitive poly(*N*-isopropylacrylamide) (PNIPAM) [78]. This resulted in the development of a CMCSG/PNIPAM cryogel characterized by a macroporous structure, achieved through free-radical copolymerization. This unique cryogel design exhibits notable advantages such as enhanced wound exudate absorption compared to traditional hydrogels. The inclusion of PNIPAM imparts exceptional temperature-responsive fluid management capabilities and effectively regulates supersaturated wound exudates. Furthermore, the amino group (–NH_2_) present in CMCS is crucial in the response to the acidic microenvironment of bacterial-infected wounds which facilitates the electrostatic capture of bacteria through self-adaptive protonation, thereby significantly boosting the antibacterial efficiency. To further enhance its therapeutic capabilities, this study introduced l-arginine-loaded MoS_2_@polydopamine (MSPA) into the CMCSG/PNIPAM cryogel, resulting in the formation of a self-adaptive NZ and nitric oxide (NO) cascade release carrier. This composite addressed diverse wound healing needs, including bacterial capture, exudate control, antibacterial therapy, self-adaptive NZ activity, and promotion of angiogenesis.

The injectability of hydrogels also plays a crucial functional role in NZ@hydrogels [79]. The ease with which the hydrogel can be injected ensures minimally invasive procedures, thereby reducing patient discomfort and recovery time. Moreover, the injectability of NZ@hydrogels facilitates their use in challenging anatomical sites that may be difficult to reach using traditional methods. For example, Bai et al. formulated an injectable hydrogel that integrates Au-Pt NZs to serve as a self-promoted cascade NZ to improve the healing process of bacterial-infected wounds [80]. Au-Pt NZs directly cross-link with 4arm-PEG-SH, eliminating the necessity for additional cross-linking agents due to the formation of coordination bonds. This unique feature imparts injectability to the resulting NZ@hydrogel. Upon application, the hydrogel transforms glucose into gluconic acid and hydrogen peroxide (H_2_O_2_), establishing a conducive environment and abundant substrate for subsequent POD-like reactions, effectively eradicating bacteria.

The mechanical properties of hydrogels can also be tuned to match the stiffness of native skin tissue, providing mechanical support for the wound area [81]. Their conformability and adhesiveness make hydrogels adhere securely to irregular wound surfaces ensuring continuous contact between NZs and wound bed. For example, Xie et al. developed a multifunctional bioadhesive hydrogel, incorporating a dual colorimetric system composed of PVA, dextran (Dex), borax, bromothymol blue (BTB), fluorescein thiocyanate (FITC), and functionalized with tungsten disulfide-catechol NZ (CL/WS_2_) [82]. Furthermore, the introduction of phenolic hydroxyl groups not only facilitated the even dispersion and stability of NZs within the hydrogels but also promoted their adhesion. This hydrogel serves as an exceptional biological adhesive, achieving consistently stronger tissue adhesion compared to commercial dressings. The real-time monitoring of wounds was possible by integrating a sensor that captures visual images of bacterial infections using a smartphone and translates them into on-site pH signals, which represents a promising system for future intelligent wound management. Similarly, Li and colleagues developed a NZ@hydrogel based on MoS_2_ NZs loaded onto carbon nanotubes (CNT) [83]. The dynamically cross-linked multifunctional hydrogel comprised PVA, sodium alginate (SA), and borax, demonstrating adhesiveness, self-healing, and shape-adaptive properties. Importantly, the shape-adaptive adhesive NZ@hydrogel effectively covered the entire wound area, optimizing the functionality of MoS_2_ NZs with maximum efficiency. The advantageous multifunctionality of the hydrogel accelerated skin regeneration by promoting collagen deposition, reducing the expression of inflammatory factors, and elevating the levels of angiogenesis factors.

In another study, Cheng et al. developed a composite hydrogel inspired by mussels by chemically coupling GelMA with dopamine motifs to create a hydrogel dressing with an improved binding affinity to moist skin surfaces [77]. GelMA hydrogels were utilized because of their distinctive photocrosslinkable and biodegradable properties, which facilitate the in situ gelation of hydrogels on skin defects. The incorporation of CeNZ resulted in ROS-scavenging capabilities, and the release of incorporated antimicrobial peptides (AMP) from the NZ@hydrogel demonstrated prompt and efficient inhibition against four representative bacterial strains, validating its intended antimicrobial effectiveness compared to the pristine hydrogels.

The tissue-specific design of hydrogels using techniques such as 3D printing facilitates the use of NZ@hydrogels for personalized therapies [84]. Hydrogels are particularly well-suited for bioprinting as they can provide the precision and customization needed to create wound dressings tailored to the specific needs of individual patients particularly in challenging wounds like diabetic ulcers or burns [85]. Recently, Chen et al. developed cerium-based MOF NZ hydrogel by 3D printing technology, specifically designed for personalized wound dressings [67]. The hydrogel primarily comprised a network formed by the Ce cross-linking of SA and a polymerized polyacrylamide network, both interwoven to establish an interpenetrating polymer network (IPN). MOF synthesis typically requires high temperatures to provide the energy needed for cleaving the oxygen–hydrogen bond of the carboxylic acid group, limiting the construction of MOF-based hydrogels through direct-ink-writing 3D printing. To overcome this, triethylamine was introduced to neutralize the carboxylic acid of the ligands, allowing ligand dehydrogenation at room temperature. Electrospray technology was also employed to ensure the structure and dimensional stability of the 3D-printed NZ@hydrogel through uniform cross-linking between Ce and SA. The 3D-printed NZ@hydrogel demonstrated unique catalytic activity against ROS and exhibited color changes dependent on glucose concentration. In vivo experiments demonstrated that this hydrogel platform has the potential to transform conventional approaches in diabetic wound management by offering rapid, efficient, and personalized treatment solutions.

In another study, Ding et al*.* introduced a method that entails accelerated gelation of a hydrogel using MoS_2_ NZ through in situ 3D bioprinting [86]. Fast gelation of the NZ@hydrogel was achieved by mixing solutions containing benzaldehyde and cyanoacetate group-functionalized dextran with MoS_2_ NZ. This unique combination resulted in a formulation suitable for use as an ink in microfluidic 3D-bioprinting. The NZ@hydrogel was specifically designed to facilitate the healing of chronic diabetic wounds by exhibiting antioxidant, photothermal, and antibacterial properties. When the NZ@hydrogel was directly applied to chronic diabetic wounds, an accelerated healing process was marked by improved wound closure, reduced oxidative stress, and eradication of bacterial infection compared to the pristine hydrogels.

The sprayability of hydrogels also offers benefits to NZ@hydrogels in contrast to traditional wound healing methods, such as ease of application, uniform distribution, and the ability to address extensive wound areas [87]. Recently, Shang et al. developed a therapeutic hydrogel spray called ACPCAH (Au/Cu_1.6_O/P–C_3_N_5_/Arg@HA), which incorporated ultrasound-responsive hyaluronic acid (HA)-encapsulated l-arginine, ultrasmall gold nanoparticles, and Cu_1.6_O NZ-co-loaded phosphorus-doped graphitic carbon nitride NS [88]. The addition of HA not only enhances the biocompatibility and stability of NZs but also facilitates targeted breakdown by hyaluronidase (HAase) in biofilms, leading to the controlled release of l-arginine and NZ to enhance bacterial interaction. This NZ@hydrogel system was designed to serve multiple functions in a sprayable form, demonstrating anti-inflammation, antibacterial action, O_2_ supply, and promotion of cell growth, all coordinated to improve the therapeutic efficacy for diabetic wounds.

In another study, Zhang et al. developed a thermosensitive NZ@hydrogel by employing Pluronic F127 hydrogels that contain flower-like Ni_3_(HITP)_2_ nanorods in promoting wound healing [89]. Temperature-sensitive hydrogels, like F127, demonstrate rapid responsiveness to changes in ambient temperature, undergoing a transition from a solution to a gel phase which renders them valuable in skin regeneration. The Ni_3_(HITP)_2_/F127 hydrogel formulation was applied to wound surfaces using an airbrush, and the body temperature-induced rapid coagulation of the thermosensitive hydrogel. Interestingly, the inclusion of Ni_3_(HITP)_2_ NZs in the F127 aqueous solution did not result in a notable change in the phase transition temperature of hydrogels. Additionally, the hydrogels exhibited excellent viscosity and injectability. Furthermore, animal experiments demonstrated that the Ni_3_(HITP)_2_/F127 hydrogel exhibited outstanding biological safety and could enhance the rate of wound healing compared to the F127 hydrogels without Ni_3_(HITP)_2_ incorporation.

In a recent study, Xiao et al. showed that the self-healing ability offered by engineered double network hydrogels combined with the enzymatic properties of NZs was also highly effective in accelerating infected skin wounds [90]. While the Ag/MoS_2_ NZs incorporated can offer antibacterial properties, the 3D porous structure of alginate-based hydrogel was shown to effectively trap the ROS, synergistically inhibiting the microbial growth within the composite hydrogel. The NZs were incorporated into the hydrogel through a physical interaction, where Ag/MoS_2_ NZs could associate with the gel via physical adsorption interactions such as van der Waals and hydrogen bonds. The alginate chains are rich in hydroxyl groups, which facilitate the formation of dynamic hydrogen bonds instilling self-healing properties to the hydrogels at room temperature. Additionally, the hydrogels exhibited high plasticity, enabling them to be shaped as desired making them suitable for applications such as hydrogel dressings.

In summary, the roles of hydrogel in the NZ@hydrogel system are multiple and highly beneficial for effective skin regeneration, by offering suitable physicochemical and bioactive properties to specific wound conditions. The features discussed here potentiate the ability of NZ, contributing to faster and more effective wound healing, making the NZ@hydrogel system a promising biomaterial platform for wound care and skin regeneration.

## Enzymatic Mechanisms and Multifunctional Roles of NZ@Hydrogels

Recently, there has been a growing interest in incorporating various NZs into biomaterials that opens new avenues for the development of multifunctional platforms with enhanced catalytic capabilities [91–93]. It is also crucial to understand the catalytic mechanisms and explore the diverse roles of NZ@hydrogels to advance their applications across various domains. The catalytic mechanisms of these hydrogels involve harnessing the inherent catalytic activities of NZs to drive specific reactions. Hence, integrating NZs into hydrogels enables precise control of catalytic activities within the hydrogel matrix [32]. The engineering of a wide range of NZs aims to achieve high catalytic efficiency, easy synthesis, cost-effectiveness, stability, and in some cases, reusability, surpassing the competencies of natural enzymes [31]. The therapeutic efficacy of NZs relies on the dual pro-oxidative and antioxidative activities both of which are valuable for various therapeutic purposes such as wound disinfection, anti-inflammatory treatments, etc.

The catalytic performance of NZs incorporated into hydrogels is affected by several factors. The choice of the NZ itself is decisive, as different types of NZs exhibit varying catalytic activity and selectivity [94]. The size, shape, and surface chemistry of NZs can also impact their catalytic performance when incorporated into hydrogels [38]. NZs with specific shapes or sizes provide enhanced catalytic activity owing to their increased surface area, which allows for more interactions with substrates [95]. The distribution of NZs within the hydrogel matrix, whether dispersed uniformly or clustered in specific regions also affects their catalytic efficiency [44]. The loading concentration of NZs within the hydrogel matrix is also a determinant [30]. The surrounding environment conditions (temperature, pH, and ionic strength) can have a profound impact on the catalytic activity of the NZs [96]. Along with NZs, the physicochemical properties of the hydrogel itself such as its porosity, stiffness, and swelling behavior can also affect the diffusion of substrates to the NZs and subsequently influence catalytic efficiency [97]. In this manner, the compatibility between NZ and hydrogel is considered important to ensure stable integration for the long-term performance of NZs within the hydrogels [96]. NZs are prone to degradation or deactivation over time, particularly under harsh environmental conditions or when exposed to certain chemicals [47]. Therefore, the choice of stabilizing agents or encapsulation methods to protect the NZs within the hydrogel is essential to maintain their catalytic activity over extended periods.

In addition to the catalytic properties, one significant advantage of NZ@hydrogels is their multifunctionality. For instance, NZ@hydrogels can possess antibacterial properties, thanks to the ROS generation that leads to the eradication of bacteria [98]. This antimicrobial activity is particularly valuable in wound healing applications where preventing infections is critical. Moreover, NZ@hydrogels can exhibit responsive behaviors to external stimuli [99, 100]. By tailoring the properties of NZs and the hydrogel matrix, researchers can design smart hydrogels that respond to specific causes such as changes in pH, temperature, or the presence of specific molecules. These stimuli-responsive hydrogels can even release encapsulated drugs or therapeutic agents in a controlled manner providing targeted therapy. The multifunctional roles of NZ@hydrogels also extend to biosensing and diagnostic applications by enhancing sensitivity and accuracy through signal amplification [101]. They can also efficiently capture and detect specific analytes enabling the development of highly sensitive and selective biosensors for medical diagnostics that monitor skin regeneration [69, 102]. Of note, NZ@hydrogels also hold great promise for skin tissue regeneration by guiding cell behaviors such as proliferation, migration, and differentiation [38, 103].

### Enzymatic Activities of NZ@Hydrogels

The specific enzymatic properties of NZ@hydrogels depend on the composition and structure of NZs [95]. Most NZs are considered to have or naturally exhibit multiple enzymatic activities [104], and the fine-tuning of structure, composition, and surface properties is essential for controlling and improving these multiple enzymatic activities [94]. The multienzymatic NZ@hydrogels are particularly effective in complex biological settings, where a range of catalytic properties are required [105]. For instance, gold (Au) NZ-based platforms display pH-switchable CAT and POD-mimic activities while Prussian blue (PB) NP-based systems display concurrent POD, CAT, and SOD activities [106, 107].

**Fig. 2:**
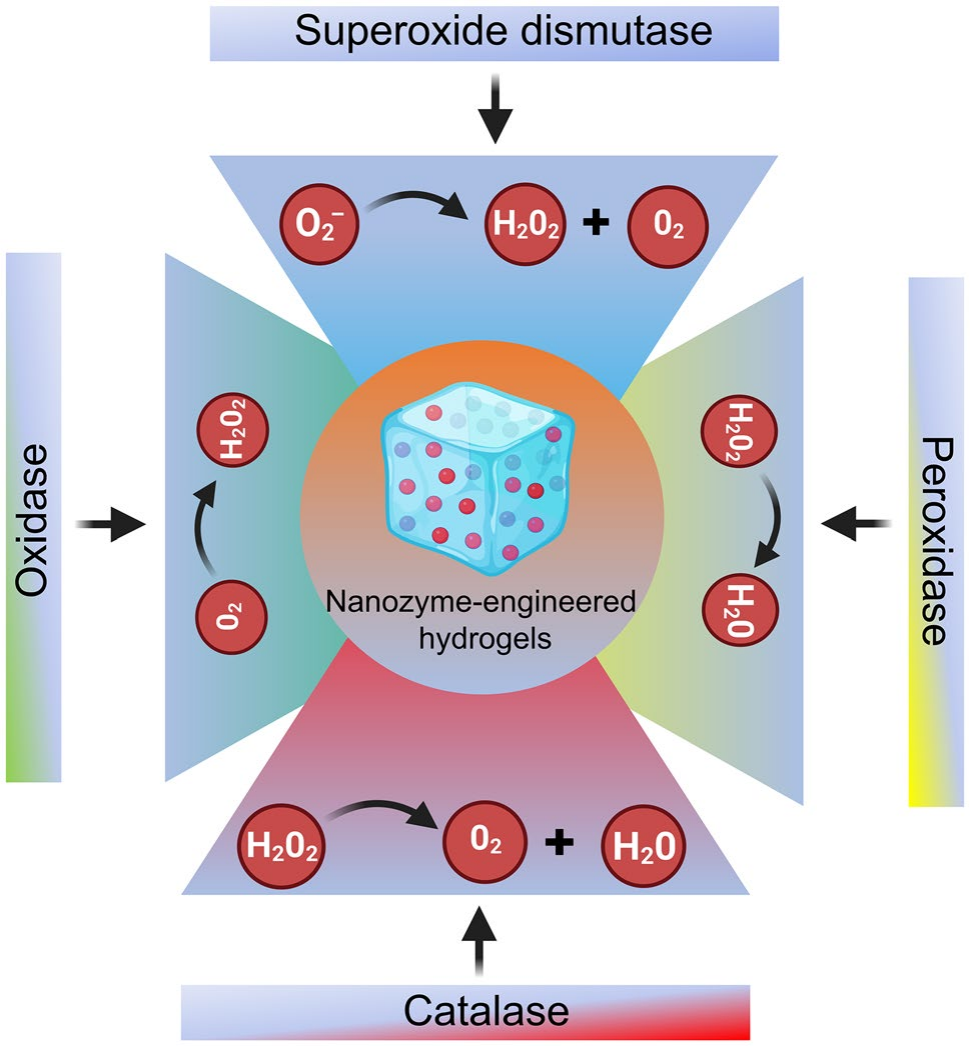
Schematic showing the chemical design of NZ@hydrogels based on intrinsic enzymatic properties

In biological systems, NZ@hydrogels possessing multienzymatic activity function in synergy. For example, many antioxidant enzymes like SOD, CAT, and glutathione peroxidase (GPx) cooperate to maintain intracellular redox equilibrium and safeguard the organism against oxidative harm and form a defense system [108]. Recognizing this, the development of hybrid platforms that resemble the intricate natural microenvironment would offer significant benefits for skin regeneration [100]. In this section, we discuss the four primary catalytic activities (POD, CAT, OXD, and SOD) demonstrated by NZ@hydrogels for skin therapy (Fig. 2).

#### Peroxidase-like Activity

Natural PODs form a diverse family of enzymes that primarily employ H_2_O_2_ to oxidize POD substrates [109]. The mechanism of POD-mimicking NZ@hydrogels involves a series of steps that catalyze the oxidation of substrates, similar to natural POD enzymes, involving substrate adsorption, H_2_O_2_ activation, substrate oxidation, and enzymatic cycle [110]. NZs in NZ@hydrogels possess a high surface area with numerous catalytic sites. Substrates such as 3,3',5,5'-tetramethylbenzidine (TMB) in many cases, adsorb onto the surface of the NZs for catalytic reactions. In the presence of H_2_O_2_, the NZs activate this molecule and lead to the generation of ROS including hydroxyl radicals (OH^•^) which initiate the POD-like reaction. The generated ROS, especially OH^•^, react with the adsorbed substrate (e.g., TMB), leading to the formation of a color product which is typically blue in case of TMB. The NZ@hydrogels are capable of efficiently performing this catalytic cycle repeatedly and hence continuously generate ROS and oxidize substrates, making them excellent POD mimics.

Subsequently, a diverse range of carbon nanomaterials exhibiting POD-like properties have been developed over time and various ranges of nanomaterials including metals, metal oxides, and metal sulfides have also been recognized for their POD-like activity [52, 111]. Fe_3_O_4_ NZs were the first to demonstrate POD-like activity and numerous Fe-based nanomaterials have already been identified to possess POD-mimicking capabilities [112]. The intrinsic ability of POD mimics that catalyze the decomposition of H_2_O_2_ into reactive OH^•^ which leads to bacterial inactivation and regulation of ROS levels has attracted considerable attention in anti-infection therapies [113].

A recent study by Zhang et al. introduced a nanoplatform named Fe@HCMS, which consisted of hollow mesoporous carbon nanospheres (HCMS) imbued with single-atom iron (Fe) [114]. The GO_*x*_ assembled on the surface of this nanoplatform leads to its demonstration of multienzyme-like activity. The hollow mesoporous structure of Fe@HCMS provided a large surface area and thereby enhanced the catalytic efficiency of the NZ while also serving as an effective platform for GO_*x*_ loading. The GO_*x*_ in turn efficiently catalyzed the conversion of glucose into H_2_O_2_ within living organisms. The presence of Fe^3+^ ions in Fe@HCMS mediated POD-like activity by converting the generated H_2_O_2_ into OH^•^ radicals which effectively deactivated bacteria. Furthermore, Fe@HCMS exhibited the capacity to deplete glutathione (GSH) which further amplifies the OH^•^ production. Additionally, a wound dressing with antibacterial properties was developed by combining this NZ with biosynthesized bacterial cellulose (BC) encased polypropylene (PP) composites for ROS-mediated biocatalytic therapy.

Recently, a growing emphasis has been put on noble metal-based NZs with POD-mimic enzymatic properties. For instance, a mussel-inspired TA-bound silver (Ag) (TA-Ag) NZ with POD-mimic property was developed by utilizing in situ reduction to form ultrasmall Ag with TA [65]. The resulting TA-Ag NZ demonstrated excellent enzymatic behavior enabling the self-setting of hydrogels. The NZ preserved a significant number of phenolic –OH groups thus establishing a dynamic redox equilibrium of phenol-quinone that conferred enduring and insistent adhesiveness to the hydrogels and facilitated its uniform distribution throughout the hydrogel network. Additionally, the NZ gifted the hydrogel with antibacterial activity through a combination of the ROS generated via POD-mimic enzymatic reactions and the inherent bactericidal properties of Ag ions.

In a similar study, Ren et al. employed an in situ growth method to synthesize Au NPs on Zeolitic Imidazolate Frameworks (ZIF-8) resulting in the development of an Au@ZIF-8 NZ [115]. Subsequently, a versatile platform for antibacterial and wound healing applications was achieved by incorporating the synthesized Au@ZIF-8 into a hydrogel composed of PVA and SA. Upon NIR laser exposure, Au@ZIF-8 exhibited high photothermal effects and improved POD-mimic activity leading to the release of zinc ions and ROS generation. These findings highlight the potential of Au@ZIF-8 in treating infected diabetic wounds through bacterial membrane interruption and promoted protein seepage.

#### Catalase-like Activity

CAT is a ubiquitous enzyme found in nearly all living organisms and is responsible for safeguarding cells against the harmful effects of H_2_O_2_ by breaking down it into H_2_O and O_2_ [116]. CAT-like NZ@hydrogels replicate the enzymatic activity of CAT and this mechanism involves several steps such as substrate binding, catalytic activation, ROS generation, and catalytic regeneration. NZs present in NZ@hydrogels have a high surface area and contain catalytic sites. When exposed to H_2_O_2_, the NZs allow the substrate to bind to their active sites, which induces a catalytic reaction, facilitating the breakdown of H_2_O_2_ into H_2_O and O_2_ [20]. During the decomposition of H_2_O_2_, ROS including superoxide radical (O_2_^•−^) and singlet oxygen (^1^O_2_) are generated as intermediates that play a crucial role in the catalytic process and are capable of reacting with other substrates or facilitating oxidative reactions. NZ@hydrogels can efficiently carry out the catalytic cycle repeatedly, thus ensuring the continuous decomposition of H_2_O_2_ into H_2_O and O_2_.

By working in tandem with SOD, CATs efficiently regulate cellular H_2_O_2_ levels and thereby allay oxidative damage. Deficiencies or dysfunctions in these enzymes are associated with various degenerative diseases [117]. Thus, different nanomaterials were explored that exhibit CAT-like activities such as nCe, iron oxides, AuNPs, and cobalt oxide NPs [118]. For instance, a multifunctional hydrogel with a multienzyme-like activity was developed by combining mussel-inspired carbon dot reduced-Ag (CDs/AgNPs) and Cu/Fe-nitrogen-imbued carbon (Cu, Fe-NC) [119]. The NZ exhibited both GSH depletion and OXD-like activity that resulted in hydrogels with exceptional antibacterial properties. Particularly in the inflammatory stage of wound healing where bacterial elimination is critical, the hydrogel exhibited CAT-like properties by catalyzing intracellular H_2_O_2_. This process supplied sufficient O_2_ that effectively alleviated hypoxia within the wound. Moreover, the inclusion of CDs/AgNPs, which contained catechol group generated hydrogels with dynamic redox equilibrium properties that resulted in adhesive characteristics that were effective in promoting wound healing and preventing bacterial infections.

**Fig. 3:**
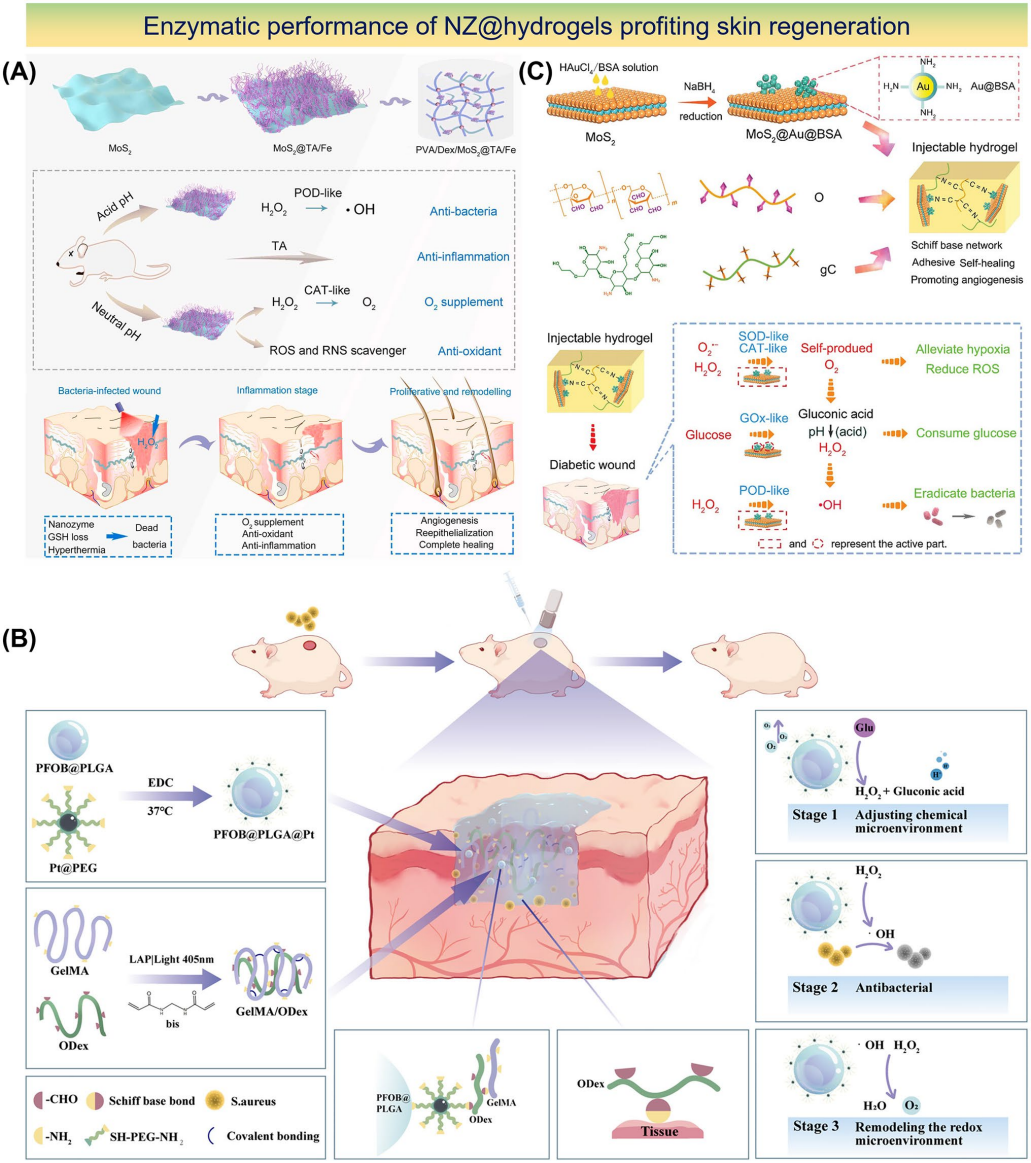
Design of NZ@hydrogels with dominant enzymatic performance profiting skin regeneration. **a** Schematic diagram showcasing the synthesis process and the dual enzyme-mimic activity of MoS_2_@TA/Fe NSs aimed at enhancing wound repair [50]. Copyright 2021, Elsevier. **b** Synthesis of PFOB@PLGA@Pt/GelMA/ODex nanohybrid double network hydrogels with exceptional enzymatic and antibacterial attributes for advancing wound healing process [127]. Copyright 2023, American Chemical Society. **c** Synthesis of MoS_2_@Au@BSA NS and the preparation of an injectable hydrogel using an O_2_-supplying glucose-powered cascade reaction for the reconstruction of infected diabetic skin [133]. Copyright 2022, John Wiley and Sons, Inc

Recently, Molybdenum disulfide (MoS_2_), a nanomaterial with NIR absorption and POD-like properties has shown promise in antibacterial treatment [120]. However, MoS_2_ alone lacks sufficient CAT-like activity for O_2_ generation. To address such drawbacks, a recent study used TA combined with Fe to form a TA/Fe complex that results in hybrid NSs of MoS_2_@TA/Fe with excellent photothermal activity. A sticky, self-repairable, and shape-adjustable hydrogel was thus formed through the dynamic boron ester bonds between MoS_2_@TA/Fe NSs, PVA, Dex, and borax [50]. The hydrogel exhibited exceptional antibacterial efficacy through PTT, GSH depletion, and POD-mimic behavior in acidic conditions (Fig. 3a). Additionally, in neutral settings, the hydrogel demonstrated CAT-mimic nature to deliver O_2_, antioxidant properties, and targeted reduction of inflammation through TA. Previous studies have also demonstrated that AuNPs exhibit remarkable GO_*x*_ activity and promote glucose consumption [121]. Similarly, platinum NPs (PtNPs) possess CAT-like activity that converts H_2_O_2_ into O_2_ and thus facilitates O_2_ release while scavenging ROS [122]. The combination of Au and Pt within nanoplatforms thus leads to synergistic effects. Based on this, self-healing hydrogels with multifunctional properties were developed to modulate the intricate microenvironment of diabetic ulcers [123]. The incorporation of Au-Pt NZs conferred GO_*x*_ and CAT-like functionality to the hydrogels which effectively reduce blood glucose levels while mitigating oxidative damage and supplying O_2_.

#### Oxidase-like Activity

OXD is an enzyme that plays a crucial role in cellular metabolism and catalyzes oxidation reactions of various substrates using O_2_ to generate ROS such as H_2_O_2_ or O_2_^•−^ [124]. The OXD-like activity of NZ@hydrogels refers to their ability to mimic the enzymatic functions of natural OXDs. The mechanism of OXD-like activity involves several key steps, such as substrate binding, O_2_ activation, substrate oxidation, and catalytic regeneration [124]. NZs in NZ@hydrogels have a high surface area with catalytic sites. When a substrate (organic compounds or molecules susceptible to oxidation) interacts with these NZs, it can bind to the active sites and thus activate O_2_, which is readily available in the surrounding environment. This activation process varies depending on the type of NZ but often involves ROS generation or electron transfer from the substrate to O_2_. The activated O_2_ or ROS reacts with the bound substrate, resulting in its oxidation and leading to the conversion of the substrate into its oxidized form with the release of oxidation by-products. NZ@hydrogels can continuously activate O_2_ or ROS and facilitate the oxidation of substrates, thereby mimicking the enzymatic function of natural OXDs.

Unlike POD-mimic reactions, OXD-like systems do not rely on pre-existing H_2_O_2_ but instead generate H_2_O_2_ and O_2_^•−^ radicals in certain cases. This unique characteristic allows OXD enzymes and NZs that mimic OXD activity to effectively convert colorless substrates into colored products by in situ generation of H_2_O_2_ and O_2_^•−^ radicals. Consequently, they serve as excellent agents for the detection of biological or chemical molecules [125]. Recently, several NZ@hydrogels were reported to exhibit OXD-like activities for various skin-related applications. However, most of them exhibit optimal activity at acidic pH conditions, which differ from the near-neutral pH found in biological systems. To address such limitations, Chen et al. developed 9-fluorenyl-methoxycarbonyl-modified diphenylalanine (Fmoc-FF) hydrogel that enhances the OXD and POD-mimic activities of PtNPs even at neutral or alkaline pH levels [126]. The Fmoc-FF hydrogel creates an acidic condition for the PtNPs through the generation of protons (H^+^) from F dissociation at neutral pH. The encapsulation of PtNPs within the Fmoc-FF hydrogel at neutral pH increased both OXD and POD-mimic activities. By synergistically harnessing the augmented enzymatic functions of PtNPs and the intrinsic antibacterial attributes of the Fmoc-FF hydrogel, the NZ@hydrogel demonstrates notable antibacterial effects that promote skin regeneration.

Another study developed a microenvironment-adaptive nanohybrid double network hydrogel utilizing a composite of PtNZ consisting of perfluorooctyl bromide (PFOB) and poly (lactic-co-glycolic acid) (PLGA) [127]. The hydrogel has two interconnected networks: the first network was formed by gelatin methacryloyl (GelMA) while the second dynamic network comprised of oxidized dextran (ODex) and PFOB@PLGA@Pt was dispersed into the hydrogel via Schiff base bonds. PFOB@PLGA@Pt provides catalytic and O_2_ supply functions and thus acts as a GO_*x*_-mimic to decrease glucose levels in the wound and regulate the pH from alkaline to acidic (Fig. 3b). By activating POD, NADH oxidase (NO_x_) and OXD-like enzymatic activities and synergistic antimicrobial effects were achieved through the production of ROS [128]. During the later stages of healing, CAT-like and SOD-like activities remodeled the redox microenvironment by effectively scavenging excessive ROS and facilitating the transition of the wound from the inflammatory to the proliferative phase. The presence of PFOB in the system also contributed to O_2_ availability for enzymatic reactions and regulated wound hypoxia. The efficacy of PFOB@PLGA@Pt/GelMA/ODex hydrogels was further demonstrated in diabetic wound repair.

#### Superoxide Dismutase-like Activity

The O_2_^•−^ radical is a primary free radical generated in mammalian cells that acts as a precursor to several other types of ROS [129]. SOD enzymes play a crucial role in effectively regulating cellular O_2_^•−^ levels. Nevertheless, any dysregulation in SOD or mutations affecting their catalytic activity can have serious consequences that potentially give rise to various skin disorders [129]. NZ@hydrogels that mimic the activity of SOD replicate the enzymatic function of catalyzing the conversion of O_2_^•−^ into O_2_ and H_2_O_2_. The mechanism of SOD mimics involves interaction with O_2_^•−^, catalytic disproportionation, H_2_O_2_ generation, and enzymatic regeneration [130]. When O_2_^•−^ are present in their proximity, these radicals can interact with the NZ surface, either through physical adsorption or by undergoing redox reactions with the catalytic sites of NZs. NZs facilitate the catalytic disproportionation of O_2_^•−^ involves the conversion of two O_2_^•−^ into one molecule of O_2_ and one molecule of H_2_O_2_. The H_2_O_2_ produced during the disproportionation of O_2_^•−^ is an important product of the catalytic reaction as it can serve as a signaling molecule and can participate in various biological and chemical processes. NZ@hydrogels can efficiently convert O_2_^•−^ into O_2_ and H_2_O_2_ and hence could mimic the essential function of SOD.

Under natural conditions, O_2_^•−^ can undergo self-decay but at an extremely slow rate. Therefore, SOD catalyzes the conversion of O_2_^•−^ anions into H_2_O_2_ and O_2_ and acts as an important antioxidant. To date, only a limited number of NZs like nCe have been known to demonstrate the O_2_^•−^ scavenging capability [131]. Extensive evidence suggests that nCe with a higher Ce^3+^/Ce^4+^ ratio on their surface exhibits SOD-like activity while a lower ratio results in CAT-like activity [132]. Although the natural SOD enzyme is essential for O_2_^•−^ metabolism, its inherent limitations such as short-term stability and high synthesis cost presents an opportunity for the development of more efficient alternatives. Inspired by the SOD-mimicking properties of nCe, a hybrid hydrogel patch containing these NPs was recently formulated to alleviate symptoms of AD [49]. The incorporation of nCe into the alginate hydrogel matrix exhibited favorable mechanical performance and biological compatibility. Moreover, it demonstrated efficient CAT and SOD-mimic behavior that provides cell-protective effects under elevated oxidative harm brought by H_2_O_2_ and 1-chloro-2,4-dinitrobenzene (DNCB). Furthermore, nCe undergoes an auto-regeneration process where Ce^4+^ is converted back to Ce^3+^ oxidation state within a few days ensuring neutralization of additional O_2_^•−^ radicals.

In another study, defect-rich MoS_2_ NSs loaded with bovine serum albumin (BSA)-modified AuNP called MoS_2_@Au@BSA NSs were incorporated into an injectable hydrogel for treating diabetic wounds [133]. The hydrogel consisted of ODex and glycol chitosan (gC) cross-linked through Schiff base chemistry. The MoS_2_ NSs demonstrated POD-mimic activity under acidic settings and SOD and CAT-mimic activity under neutral settings. These enzymatic reactions convert detrimental compounds like O_2_^•−^ and H_2_O_2_ into harmless substances thereby reducing oxidative stress and improving wound healing process. Moreover, hydrogel facilitates a self-supplying cascade reaction of O_2_. The incorporation of BSA in NPs enhances the activity of multiple enzymes while AuNPs act as catalysts like GO_*x*_ that promote glucose oxidation and bacterial elimination (Fig. 3c). In short, MoS_2_@Au@BSA mimic SOD behavior in alkaline wound pH and mitigates oxidative stress, alleviates hypoxia, and accelerates the healing of diabetic wounds. Lately, hydrogels incorporated with Metal–organic framework (MOF)-based NZs were developed to address disorders resulting from imbalanced O_2_^•−^ levels. For instance, Zhang et al. reported well-structured Ni-MOF) nanorods called Ni_3_(2,3,6,7,10,11-hexaiminotriphenylene)_2_ (Ni_3_(HITP)_2_) nanorods [89]. These nanorods possessed excellent conductivity, defined structures, numerous catalytic sites, and demonstrated SOD-like enzyme activity that effectively scavenged various free radicals. Additionally, these platforms promoted fibroblast migration, angiogenesis, and polarization of macrophages to anti-inflammatory (M2) phenotype. Later, an injectable and sprayable therapeutic formulation was made by encapsulating Ni_3_(HITP)_2_ MOF nanomaterials within temperature-sensitive Pluronic F127 hydrogels offering a promising solution for chronic skin wounds.

### Tuning the Enzymatic Properties of NZ@Hydrogels

Despite the substantial growth made in the development of various NZ-engineered platforms for biomedical applications, understanding the fundamental factors that influence their catalytic performance remains a challenge [20, 134]. This arises from the intricate interplay between the inherent structure of NZs and the external environment in which they function. Additionally, understanding the catalytic mechanisms is decisive for the rational design of novel NZs with inherent catalytic abilities as it has been extensively utilized in biomedicine as a controllable and multifunctional platform [108, 135].

The effectiveness of NZ@hydrogels in catalyzing reactions is greatly influenced by the nanostructure of NZs present [95]. Controlling the size, morphology, composition, and surface characteristics of NZs can modify their catalytic activity and specificity [135]. For example, Gao et al. investigated the POD-like activities of spherical Fe_3_O_4_ NZs of different sizes (300, 150, and 30 nm) and observed an increase in POD-like activity for smaller size compared to larger size owing to the higher surface-to-volume ratio of smaller NZs [23]. In another study, Luo and colleagues examined the GO_*x*_-mimicking nature of AuNPs with varying sizes (13, 20, 30, and 50 nm) at an equivalent concentration and observed an enhanced GO_*x*_-like activity due to the smaller size of NZs [121]. Similarly, Korsvik et al. observed that CeNZs with a size greater than 5 nm exhibit relatively low Ce^3+^/Ce^4+^ ratios, resulting in weaker SOD-mimic activity compared to smaller CeNZs with a size of 3–5 nm [136]. Baldim et al. also demonstrated that the catalytic activity of CeNZs relies on the concentration of Ce^3+^ on its surface [132]. The studies on the enzyme-mimicking capabilities of various CeNZs with distinct specific surface areas and differing Ce^3+^ fractions indicated enhanced catalytic effects in smaller particles with higher Ce^3+^ fraction.

To investigate the impact of particle morphology on the multienzymatic performance of NZs, Singh et al. synthesized Mn_3_O_4_ NZs with different morphologies, such as flower-like, flake-like, hexagonal plates, polyhedrons, and cubes [137]. They subsequently evaluated their catalytic activities in mimicking SOD, CAT, and GP_x_. The flower-like Mn_3_O_4_ NZs exhibited the highest SOD, CAT, and GP_x_-like activities, followed by the flake-like Mn_3_O_4_ NZs. Notably, the specific surface area of the flower-like Mn_3_O_4_ NZs surpassed that of the other morphologies. In another study, Ghosh et al. synthesized four distinct types of vanadium pentoxide (V_2_O_5_) NZs, each possessing varied morphologies, such as nanowires, nanosheets, nanoflowers, and nanospheres [138]. These morphologies exhibited specific facets and hence the reactivity of the four V_2_O_5_ NZs with H_2_O_2_ varied and the nanospheres displayed significantly higher GP_x_-like activity compared to the others.

Another strategy is the incorporation of specific ligands or molecules on the surface of NZs, which can alter their catalytic properties [139]. These surface modifications can be achieved through functionalization with organic molecules, polymers, or biomolecules that allow for precise control over the catalytic behavior of the NZs when incorporated in hydrogel matrices by enhancing their selectivity for specific substrates. For example, Fan et al. enhanced the POD-mimic activity of Fe_3_O_4_ NZs by multiple-folds through the attachment of histidine [140]. It was observed that amino acid modification alone can significantly improve the catalytic properties of the Fe_3_O_4_ NZs, replicating the structural features of the active site found in natural horseradish peroxidase (HRP). Beyond its impact on POD-like activity, the histidine modification also improves the CAT-like activity, indicating an enhanced affinity for H_2_O_2_ at the initial reaction stage. In another study, the POD-like activity of gold nanozymes (GNZs) toward glutathione was improved by introducing two amino acid residues as active sites on the surface [141]. Selenocysteine, recognized as the active site in natural GPx, exhibits the ability to adsorb H_2_O_2_. To achieve this, a synthetic pentapeptide (Ser-Arg-Gly-Asp-Cys) with SH-bonding capability is self-assembled on the surface of GNZs. This facilitates the bonding of selenium with the thiol head of glutathione, serving as the active site. Moreover, a second active site featuring cysteine is self-assembled on GNZ to absorb anionic substrates using its amine group. The integration of these two active sites leads to a remarkable increase in POD-mimic activity on modified GNZs compared to their unmodified ones. Similarly, Liu et al. demonstrated the exceptional enzymatic properties achieved by conjugating CeNZs with the cage protein apoferritin [142]. This enhanced performance was attributed to a charge transfer at the interface of the protein corona and CeNZs, leading to an increased proportion of Ce^3+^. The resulting complex of NZ-cage protein functioned as an artificial redox enzyme with excellent SOD-mimetic activity.

Opting for suitable structure-oriented agents during synthesis can also be an effective approach to enhance the catalytic activity of NZs. For example, Hao et al. confirmed this by developing Cu_*X*_O-Ph NZs utilizing phenylalanine (Phe) as a structure-directing agent, demonstrating favorable biocompatibility and possessing multiple antioxidant enzyme-like properties [143]. This observation revealed that the choice of diverse molecular structure-oriented agents led to variations in the shapes and activities of the NZs.

Single-atom catalysis also serves as another key approach for improving the catalytic activity of NZs [144]. For instance, Yan et al. functionalized single-atom Pt on ultrasmall nCe clusters, resulting in a multiple-fold increase in ROS scavenging activity compared to CeNZ [145]. The improved Ce^3+^/Ce^4+^ ratio in Pt/CeNZ and the catalytically active sites provided by single-atom Pt with O_2_ vacancies were identified as the primary factors contributing to the enhanced catalytic activity. Similarly, Wang et al. utilized Mn_3_[Co(CN)_6_]_2_ MOFs as supporting material to incorporate a single atom of Ru [146]. Here Ru functioned as a catalytic site for endogenous O_2_ production, partly replacing Co due to its stronger coordination with the ligand terminal carbon. The exceptional CAT-like activity of this NZ stemmed from unsaturated Ru–C coordination sites, facilitating the rapid H_2_O_2_ decomposition.

In addition to surface modification and structural optimization, the catalytic activities of NZ@hydrogels can also be tuned through external stimuli-responsive mechanisms [99]. Smart NZs that respond to specific stimuli, such as pH, temperature, or light, are incorporated into hydrogels which can switch their enzymatic activity on or off in response to changes in their environment [147]. For instance, Yang et al. developed NZ-engineered cryogels that exhibit responsiveness to multiple stimuli, including pH, NIR light, and temperature that effectively eliminate methicillin-resistant staphylococcus aureus (MRSA) bacterial biofilms through a combination of nitric oxide (NO)-assisted photodynamic and PTT, making them suitable for addressing various stages of infected wound healing [78]. The platform was made by integrating MoS_2_ and l-arginine (MSPA), a nitric oxide (NO) release precursor, into cryogels formed from carboxymethyl chitosan/poly(N-isopropylacrylamide) (CMCS/PNIPAM). These cryogels exhibit sensitivity to a range of stimuli, resulting in the development of versatile antibacterial materials tailored for different stages of infected wound healing. In a slightly acidic bacterial microenvironment, the cryogels demonstrate enhanced bacterial capture capabilities through acid-triggered protonation behavior, thereby significantly improving the efficiency of photodynamic antibacterial processes. The cryogels enable precise, on-demand release of ROS and NO, along with the capability to remotely control infected biofluid through NIR light activation as a trigger switch. Most importantly, these platforms demonstrate efficacy in reducing wound infection in vivo by alleviating oxidative stress and accelerating collagen deposition and angiogenesis. This highlights the potential of multiple stimuli-responsive self-adaptive wound dressings as a promising approach for treating infected wounds. Additionally, the incorporation of such smart NZs can provide on-demand control over the NZ activity.

Another emerging approach for tuning the catalytic activities of NZ@hydrogels involves using hybrid NZs [148]. Hybrid NZs are designed by combining multiple types of nanomaterials, each with distinct catalytic properties to create synergistic effects [149]. This approach enables the development of NZs with enhanced catalytic activities, stability, and specificity. The combination of metallic NZs with carbon-based nanomaterials or inorganic nanomaterials has been shown to create hybrid NZs with enhanced catalytic activity [150]. For instance, Xi et al. revealed metal valence state-dependent catalytic properties in metal–carbon-based hybrid NZs [151]. These NZs, composed of copper (Cu) and carbon, exhibited POD, CAT, and SOD-like activities, based on the valence state of Cu (Cu^0^ to Cu^2+^). Importantly, they also displayed valence state-dependent antibacterial properties. The CuO-modified hybrid NZs damaged bacterial membranes and caused DNA damage in gram-negative bacteria, while Cu-modified hybrid NZs generated ROS-like PODs, targeting both gram-positive and gram-negative bacteria. In vivo experiments with bacteria-infected animal models validated their antibacterial efficacy. Additionally, many hybrid NZs were reported where noble metal NPs are combined with other catalytic nanomaterials such as MOFs [25]. This approach offers synergistic effects and enables further tuning of the catalytic properties to achieve the desired functionality in hydrogels.

In another study, Huang et al. combined V_2_O_5_ and MnO_2_ NZs via dopamine to create a synergistic antioxidant system with multiple enzyme-mimicking activities to mimic an intracellular defense system [152]. The V_2_O_5_ nanowire was employed as a mimic for GPx, while the MnO_2_ functioned as SOD and CAT mimic. The self-assembly of these NZs was facilitated by using dopamine as a linker. Apart from the antioxidant functions exhibited by NZs, the combination of nanocomposites with dopamine resulted in synergistic antioxidative effects. The resulting V_2_O_5_@pDA@MnO_2_ composite served as a multifunctional NZ platform, imitating the collaborative intracellular antioxidant enzyme defense mechanism involving SOD, CAT, and GPx. The MnO_2_ catalyzed the conversion of O_2_^•−^ to O_2_ and H_2_O_2_ and further transformed it into H_2_O while V_2_O_5_ efficiently catalyzed the conversion of H_2_O_2_ to harmless byproducts. The in vitro experiments validated the biocompatibility of these platforms, showcasing their outstanding capability to remove intracellular ROS and protect cellular components from oxidative stress. Significantly, within an in vivo inflammation model, the nanocomposites exhibit a gradual reduction in ROS levels, highlighting their potential for applications in inflammation therapy.

Similarly, Zhang et al. observed a significant enhancement in the POD-like activity of *γ*-Fe_2_O_3_ NZs coated with PB [153]. By incorporating PB NZ and leveraging its excellent electrochemical behavior and catalytic properties, coupled with the high stability and superparamagnetism of *γ*-Fe_2_O_3_, the resulting nanocomposites could potentially exhibit both superparamagnetism and POD-like activity. The magnetic properties and POD-mimic activity of resulting NZs were thoroughly assessed in the study and it was observed that even with an increased PB content, the magnetic characteristics maintained a consistently high level. The POD-like activity exhibited improvement with a higher proportion of PB, following Michaelis–Menten kinetics. The computed kinetic parameters revealed a robust substrate affinity and exceptionally elevated catalytic activity, surpassing that of Fe_2_O_3_ NZs of comparable size by multiple folds. Utilizing their strong catalytic capabilities, the hybrid NZ modified with PB was subsequently bound to staphylococcal protein through electrostatic adsorption, confirming their potential utilization in enzyme immunoassays.

Furthermore, recently researchers started exploring the use of artificial intelligence (AI) and machine learning algorithms to optimize the catalytic activities of NZs [154]. For example, Wei et al. introduced a data-driven approach that employs machine-learning algorithms to comprehend particle–property relationships, enabling the classification and quantitative prediction of enzyme-like activity displayed by NZs [155]. These models are successfully applied to predict or design NZs with desired enzymatic properties by unveiling the unobserved relationship between different periods of transition metals and their enzymatic performance. In another recent study, Zhang et al. identified the SOD-like properties of manganese thiophosphite (MnPS_3_) NZ through the application of ML tools offering a general framework for the expedited discovery of SOD-like NZs for the rational design of next-generation NZs [156]. Similarly, Li et al. also proposed an approach for the design of hydrolytic NZs, contributing to the expansion of NZ diversity and offering insights into the potential future advancements in NZ engineering [157].

By utilizing computational modeling and data-driven approaches, it is possible to predict and design NZs with precise catalytic properties tailored to the requirements of specific applications [155]. Moreover, such approaches allow for the rapid development of NZs that are highly efficient, cost-effective, and customizable for various therapeutic and diagnostic purposes. Collectively, the precise control of the catalytic properties of NZs opens exciting possibilities for engineering hydrogels with tailored functionality. Through careful design and tuning of NZs, we can harness their catalytic activity to enhance the performance of hydrogel materials in an extensive range of therapeutic applications, particularly skin regeneration. The list of NZ platforms utilized to engineer hydrogels and their key catalytic properties is summarized in Table 1.

**Table 1:** List of various NZ platforms utilized to engineer hydrogels and their key catalytic properties analyzed

Classification	Nanozyme	Analyzed catalytic property	References
Ce-based	nCe	SOD, CAT	[49]
	CeNZ	SOD, CAT	[77]
	Ce-MOF	SOD, CAT, GO_*x*_	[67]
	CeNZ	SOD, CAT	[45]
Mn-based	MnO_2_	CAT, POD	[158]
	MnCoO	CAT	[159]
	GO_*x*_-MnO_2_	POD, GO_*x*_	[160]
	EPL-MnO_2_	SOD	[41]
Fe-based	Fe@HCMS/GO_*x*_	POD, GO_*x*_, GSH	[114]
	Mica-Fe_3_O_4_	POD	[63]
	Fe_3_O_4_@GO	POD, SOD	[161]
	Fe-MIL-88NH_2_	POD, OXD	[162]
	FePO_4_	POD, SOD, CAT	[64]
	GA/Fe@AP (GAP)	CAT	[163]
	Fe_2_O_3_-GO_*x*_@ZIF-8	GO_*x*_, CAT	[164]
Mo-based	MoS_2_@TA/Fe	POD, CAT	[50]
	CNT@MoS_2_	POD, SOD, CAT	[83]
	MoS_2_	CAT	[86]
	Ag/MoS_2_	POD	[90]
	MoS_2_@Au@BSA	GO_*x*_, POD, CAT, SOD	[133]
	MoS_2_	POD	[165]
	MoS_2_-PDA	CAT	[166]
	MoNP	POD, SOD, CAT	[167]
MOF-based	MOF-818	SOD, CAT	[75]
	Ni_3_(HITP)_2_ MOF	SOD	[89]
	MOF(Fe–Cu)/GO_*x*_	POD, GO_*x*_	[168]
	Zr-Fc MOF	POD	[169]
Cu-based	Au/Cu_1.6_O/P–C_3_N_5_/Arg NSs	SOD, CAT, POD, GO_*x*_, NOS	[88]
	Cu_5.4_O	SOD, CAT, GP_*x*_	[170]
	Cu_2_O/Pt	POD, GO_*x*_,	[171]
	Cu_2_MoS_4_	POD	[172]
	Ni_4_Cu_2_	SOD, CAT	[51]
Au-based	Au-Pt	POD	[80]
	Au@ZIF-8	POD	[115]
Ag-based	PDA-AgNPs	POD	[56]
	TA-Ag NZ	POD	[65]
W-based	WS_2_/CL	CAT	[82]
PB-based	PB NZ	SOD	[42]
	PB NZ/GO_*x*_	POD, GO_*x*_	[44]
Pt-based	PtNZ	OXD, POD, GO_*x*_, NO_*x*_, SOD, CAT	[127]

### Multifunctional Roles of NZ@Hydrogels

**Fig. 4:**
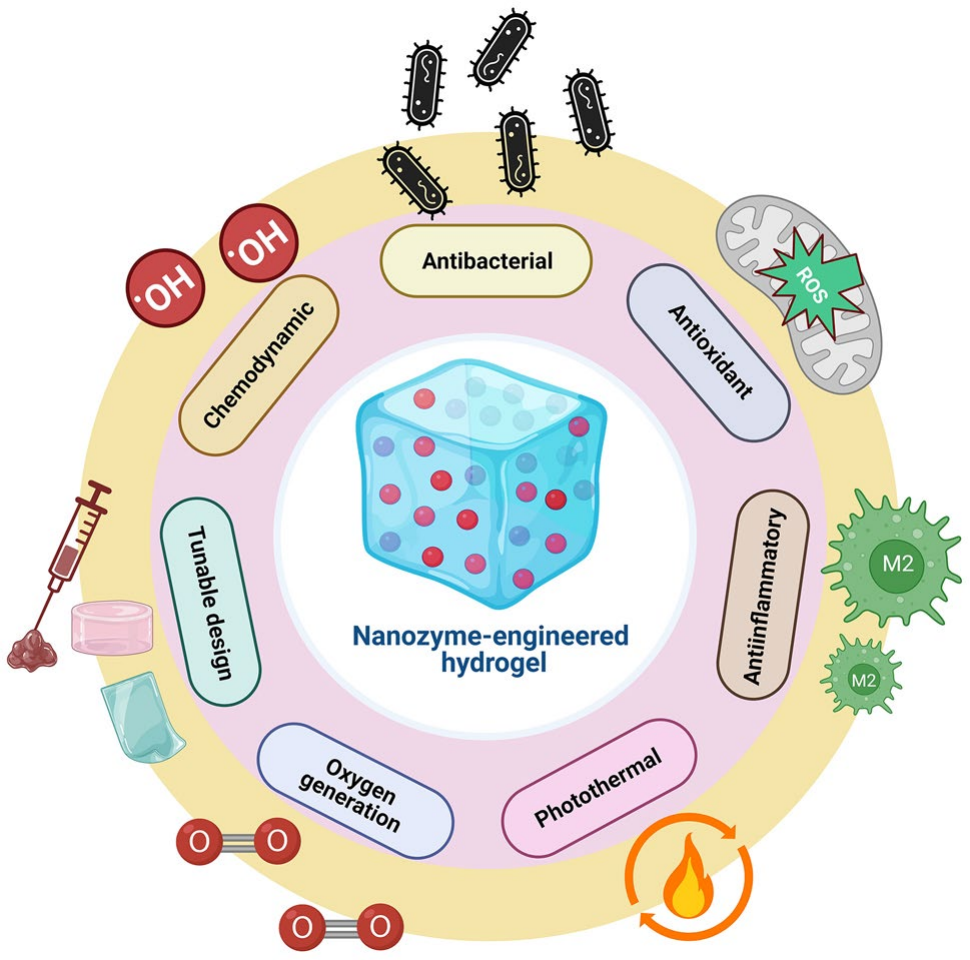
Schematic showing the multifunctional roles of NZ@hydrogels for skin therapy

NZ@hydrogels represent a promising strategy to support various aspects of skin regeneration by combining the distinctive characteristics of hydrogels with the catalytic capacity of NZs [32]. An essential function involves the regulation of ROS, which can reduce oxidative stress and accelerate the wound healing process [47]. Moreover, NZ@hydrogels can address the challenge of hypoxia by serving as an O_2_ delivery system. Through catalytic reactions, the hydrogels generate O_2_ or convert ROS into harmless substances that improve O_2_ availability and promote angiogenesis, cell proliferation, and migration [173]. The controlled release of bioactive factors is another advantage of these materials. Through the entrapment of growth factors, cytokines, or therapeutic agents within the hydrogel matrix, a controlled and prolonged release mechanism is established which enhances the therapeutic potential that ultimately maximizes their effectiveness in promoting skin regeneration [174, 175]. Additionally, NZ@hydrogels can exhibit antimicrobial properties that inhibit bacterial growth and prevent infections in chronic wounds [176]. The integration of specific NZs within the hydrogel matrix can also modulate cellular behaviors such as fibroblast migration, angiogenesis, and immune cell polarization, which further supports the healing process [47]. In essence, NZ@hydrogels present a well-rounded answer for skin regeneration by effectively dealing with issues like oxidative stress, hypoxia, inflammation, antimicrobial protection, and cellular modulation. Figure 4 illustrates the multifunctional role of NZ@hydrogels tailored for specialized applications in anti-inflammation and skin regeneration. The multiple functions include antibacterial, antioxidant, anti-inflammatory, photothermal, oxygen generation, tunable design, and chemo-dynamic properties. Most NZ@hydrogels demonstrate synergistic effects by integrating one or more of these specific properties. Table 2 presents a summary of various NZ@hydrogel platforms and their analyzed catalytic properties with targeted functionalities for skin therapy, with a particular focus on diabetic wound healing and infected wound healing applications.

**Table 2:** Summary of various functional NZ@hydrogel platforms and their specific catalytic properties analyzed for skin therapy

Function	Hydrogel	Nanozyme	Analyzed catalytic property	Application	References
Antioxidant	PVA/SA	CNT@MoS_2_	POD, SOD, CAT	Infected wound healing	[83]
	Gelatin	MoNP	POD, SOD, CAT	Diabetic wound healing	[167]
	PLGA-PEG-PLGA	MOF-818	SOD, CAT, GP_*x*_	Diabetic wound healing	[75]
Antibacterial	HG	FePO_4_	POD, SOD, CAT	Infected wound healing	[64]
	GM-DC	Fe-MIL-88NH_2_	POD, OXD	Infected wound healing	[162]
	Silk fibroin	Fe_3_O_4_	POD	Infected wound healing	[63]
Anti-inflammation	HMP	MnO_2_	CAT, POD	Infected diabetic wound healing	[158]
	Hep-PEG	Cu_5.4_O	SOD, CAT, GP_*x*_	Diabetic wound healing	[170]
	PAM	MOF(Fe–Cu)/GO_*x*_	POD, GO_*x*_	Infected wound healing	[168]
Photothermal therapy	Carrageenan	Zr-Fc MOF	POD	Infected wound healing	[169]
	PVA/Dex	MoS_2_@TA/Fe	POD, CAT	Infected wound healing	[50]
	MeHA	Fe_3_O_4_@GO	POD	Bacterial biofilm infection therapy	[161]
Chemo-dynamic therapy	Chitosan thermogel	CuO_2_ nanodots	GSH, GP_*x*_	Melanoma therapy and infected wound healing	[207]
	Pluronic F127	TMB/Fe^2+^/GO_*x*_	GO_*x*_	Infected diabetic wound healing	[209]
	ALG-HA	Cu_2_O/Pt nanocubes	POD, GO_*x*_,	Infected wound healing	[171]
	Poloxamer F127	Cu_2_MoS_4_	POD	Infected burn wound healing	[172]
Oxygen generation	HA-HYD	MnCoO	CAT	Diabetic wound healing	[159]
	HA encapsulated l-arginine	Au/Cu_1.6_O/P–C_3_N_5_/Arg NSs	SOD, CAT, POD, GO_*x*_, NOS	Diabetic wound healing	[88]
	ODex/gC	MoS_2_ NSs	SOD, CAT, POD	Diabetic wound healing	[133]

#### NZ@hydrogels as Antioxidants

Oxidative stress primarily arises from an imbalance between the production of ROS and the capacity of the body's antioxidant defense system [177]. This often leads to pathological conditions like inflammation and delayed wound healing. NZ@hydrogels can promote skin regeneration by counteracting oxidative stress [178]. Another merit of NZ@hydrogels is their ability to stimulate the synthesis of ECM. Oxidative stress can disrupt ECM synthesis thus hampering the wound healing process and skin regeneration. However, the antioxidant properties of NZ@hydrogels help to maintain the integrity of the ECM by facilitating the synthesis of collagen and other essential ECM components of skin [179].

For example, Li et al. considered a multifunctional hydrogel (PSCMo) synthesized using polyvinyl alcohol (PVA), sodium alginate (SA), and borax through chemical boron-ester bond cross-linking incorporated with CNT@MoS_2_ with triple enzymatic activities to eradicate bacteria and to simulate the antioxidant defense system and thereby enhancing wound repair [83]. The distinctive cross-linking mechanism present in hydrogels resulted in outstanding self-healing capabilities. The MoS_2_ NZ in the hydrogel displayed enhanced POD, CAT, and SOD-like activities, which were further improved by the inclusion of CNT and NIR irradiation. In addition to preventing infections and scavenging free radicals, the hydrogel expedited wound healing and facilitated the regeneration of skin defects infected with Staphylococcus aureus. In vivo experiments demonstrated that the NZ-hydrogel surpassed the pristine hydrogel, promoting skin regeneration by reducing inflammation, scavenging ROS, and supporting angiogenesis during the wound repair phase.

Additionally, a hydrogel based on molybdenum (Mo) was developed with intrinsic antioxidant properties. Molybdenum nanoplates (MoNP) at a low-valence state were incorporated into the gelatin-based hydrogel matrix to address the challenges associated with diabetic skin wounds by effectively scavenging harmful ROS [167]. The Mo hydrogel rapidly decreased in viscosity and elasticity at body temperature, thereby aiding effective wound filling and the release of MoNP. In vivo experiments reveal that the Mo hydrogel accelerated the wound healing process by increasing c-Myc expression. Consequently, this enhanced the expression of diverse downstream growth factors, promoted blood vessel regeneration, and collagen deposition, and accelerated cell migration. It is noteworthy that Mo is an essential element for all living organisms, which signifies the potential of this NZ@hydrogel for clinical translations.

In another study, Chao et al. formulated an efficient antioxidative platform for the healing of chronic diabetic wounds by incorporating SOD and CAT-mimic MOF-based NZ into thermosensitive PLGA-PEG-PLGA formed by a ring-opening copolymerization of d, l-lactide, and glycolide [75]. The hydrogel acted as a delivery system for MOF, ensuring continuous treatment at wound sites. The composite hydrogel enabled continuous scavenging of ROS within the wound bed and modulated the oxidative stress microenvironment from the inflammatory to proliferation phase during chronic diabetic wound healing. In contrast to the daily use of a clinical drug HEGFG, a single application of MOF/gel showed comparable effectiveness, indicating its great potential in clinical applications. Together, the NZ@hydrogels offer a promising antioxidant platform with the ability to regulate redox balance effectively. Their stability, long-lasting properties, and tunable characteristics make them highly versatile for addressing various challenges related to skin and other therapies targeting oxidative stress-related skin disorders.

#### NZ@hydrogels with Antibacterial Functions

Skin serves as a robust mechanical barrier comprised of densely packed epithelial cells that effectively prevent the invasion of foreign pathogens [180]. Nevertheless, when a wound occurs the skin becomes vulnerable to bacterial infections [181]. Although H_2_O_2_ is frequently employed for wound disinfection owing to its extensive antimicrobial capabilities, its elevated concentration can be detrimental and impede the wound healing process [182]. Hence, NZ@hydrogels are being suggested as a means to augment the regeneration of infected wounds [38]. The engineered hydrogel platforms can directly eliminate bacteria and disrupt bacterial biofilms by releasing reactive species including H_2_O_2_, which eventually promotes wound healing. Additionally, they moderate inflammation and enable the recruitment of immune cells to the wound area.

For example, an iron phosphate NZ-hydrogel (FePO_4_-HG) with specific characteristics such as a positive charge and macro-porous structure was designed to combat bacterial infections in skin defects. Notably, FePO_4_-HG demonstrated POD-mimic activity in the acidic microenvironment associated with bacterial infections [64]. It also exhibited synergistic effects resembling SOD and CAT-like activity under neutral or weak alkaline conditions. This dual-enzymatic behavior protected ordinary tissues from damage instigated by the POD activity with exogenous H_2_O_2_. Consequently, FePO_4_-HG unveiled admirable antibacterial efficacy against methicillin and ampicillin-resistant bacterial strains aided by H_2_O_2_. Importantly, the FePO_4_-HG + H_2_O_2_ system efficiently disrupted bacterial biofilm occurrence and facilitated the oxidation of GSH, which resulted in rapid bacterial death with low cellular toxicity. Animal experiments also confirmed that the FePO_4_-HG + H_2_O_2_ group effectively eliminated infection while ministering the wound healing process compared to the pristine hydrogel.

One recent study reported a novel wound dressing called CSG-M_*X*_ (where *x* represents the concentration of NZ) which consists of Fe-MIL-88NH_2_ NZ grafted onto glycidyl methacrylate modified dialdehyde chitosan (GM-DC) employing Schiff base chemistry [162]. Acryloyl Pluronic 127 (PF127-DA) was used as a cross-linking agent to create NZ-incorporated cryogels. The CSG-M_*X*_ possessed enhanced antibacterial and biocompatible properties, which was effective for managing biofluids and treating wound infections. It exhibits high hydrophilicity, positive charge, pH-responsive release, remarkable enzyme-like activity, and generates OH^•^ and O_2_^•–^ radicals. The CSG-M_*X*_ intelligently adapts to trap and destroy bacteria by leveraging factors such as bacterial potential, infection-induced pH changes, and controlled release of NZs regulated by pH. In another study, Li et al. developed a NZ@hydrogel using injectable silk fibroin/ZnO NPs/mica-Fe_3_O_4_ (SFZM) which displayed enhanced mechanical properties, spontaneous H_2_O_2_ generation and POD-mimicking nature [63]. Notably, ZnO NPs produced H_2_O_2_ without any external stimuli or light. When exposed to a weakly acidic environment, the H_2_O_2_ generated by the ZnO NPs was in situ transformed to free radicals by 2D magnetic mica-Fe_3_O_4_ NSs resulting in high antibacterial activity. Additionally, the hydrogel exhibited low rates of NZ leaching, confirming the biocompatibility in both in vitro and in vivo. Notably, ZnO sustained H_2_O_2_ generation for a minimum of 12 days, underscoring the prolonged antibacterial efficacy of the hydrogel. Importantly, the SFZM NZ hydrogel demonstrated promising in vivo adhesion and hemostasis properties that lead to accelerated healing of bacteria-infected wounds as compared to the pristine silk fibroin hydrogel.

#### NZ@hydrogels for Anti-inflammation

In normal conditions, inflammation, a natural immune response, is essential for safeguarding the body and is usually resolved by eliminating immune cells, debris, and apoptosis [183]. However, uncontrolled inflammation can cause tissue damage and give rise to various disorders [184]. Current anti-inflammatory treatments mainly rely on steroidal or nonsteroidal pharmaceuticals like nonsteroidal anti-inflammatory drugs (NSAIDs) [185]. Nevertheless, these drugs are generally considered moderately effective and have limitations such as off-target biodistribution, poor bioavailability, and overcoming biological barriers [186]. NZ@hydrogels can address this issue by acting as an alternative to drugs for anti-inflammatory strategies [72, 187]. By regulating the recruitment and activation of immune cells these hydrogels can enhance the influx of anti-inflammatory immune cells such as regulatory T cells, which simultaneously suppresses the activation of pro-inflammatory immune cells [188]. This immune modulation contributes to the restoration of immune homeostasis that leads to reduced inflammation and balanced immune responses, which ultimately supports the regeneration of inflamed skin.

Recently, a composite hydrogel was developed for this purpose while sequestering chemokines and scavenging ROS [170]. The hydrogel consisted of amine-modified star-shaped PEG (starPEG) and heparin (Hep) which contains ultrasmall NZs based on copper (Cu_5.4_O). The hydrogel demonstrated effective adsorption of inflammatory proteins found in wound fluids specifically interleukin-8 (IL-8) and monocyte chemoattractant protein-1 (MCP-1) that results in reduced migratory activity of inflammatory cells and prevents further activation of inflammatory signaling. Additionally, the hydrogel scavenged ROS present in the wound bed and thereby mitigates oxidative damage. This approach does not aim to completely suppress inflammation. Instead, it seeks to strike a balance, enabling the immune system to combat pathogens while avoiding undesirable side effects. In vivo models representing both acute normal wound healing and delayed diabetic wound healing were employed to assess the overall pro-regenerative effect of Cu_5.4_O@Hep-PEG hydrogels. It was observed that several proteins related to collagen synthesis are upregulated by Cu_5.4_O@Hep-PEG treatment compared to the pristine hydrogel. Also, the constant release of Cu_5.4_O from the hydrogel facilitated angiogenesis and promoted the formation of new blood vessels. The Cu_5.4_O@Hep-PEG was also proven to surpass the standard-of-care product Promogram in acute and chronic wounds mainly by reducing inflammation while stimulating epidermis regeneration, vascularization, and wound closure.

Tu et al. developed another versatile hydrogel with multiple functions to combat inflammation by cross-linking hydrophilic poly(PEGMA-co-GMA-co-AAm) (PPGA) polymers with manganese dioxide (MnO_2_) NZs modified with hyperbranched poly-l-lysine (HBPL) [158]. The hydrophilic polymer, poly(PEGMA-co-GMA-co-AAm) (PPGA) was made through a radical polymerization process using PEGMA, GMA, AAm, and pravastatin sodium was loaded into the hydrogel resulting in a NZ@hydrogel termed HMP. The incorporation of HBPL significantly improved the stability of MnO_2_ NZs in physiological environments. The redox catalytic properties of the MnO_2_ NZs originate from the coexistence of multivalent Mn ions, including Mn^4+^, as well as Mn^3+^ and Mn^2+^ resulting from oxygen vacancies. Interestingly, HBPL-crosslinked hydrogel exhibited high effectiveness against MRSA, E. coli, and Pseudomonas aeruginosa. In vivo studies confirmed that the HMP hydrogels exhibited reduced ROS and alleviated inflammation with a decrease in the infiltration of neutrophils and an enhanced polarization of macrophages toward the M2 phenotype. Moreover, a lower expression of inflammatory factors and chemokines were observed along with enhanced anti-inflammatory cytokine levels. Additionally, the hydrogel stimulated the release of transforming growth factor beta (TGF-β) which promoted neovascularization and collagen deposition, which resulted in thicker skin and improved epithelial structure.

Currently, most wound dressings are submissive and cannot respond to the wound microenvironment, which hinders their ability to control inflammation in skin wounds, leading to suboptimal therapeutic outcomes [189]. Recently, Zhu et al. formulated an intelligent wound dressing by combining gelatin modified with dopamine (Gel-DA) and Cu-loaded polydopamine (PDA) nanoparticles (CuPDA NPs) with HA functionalized with phenyl boronate acid (HA-PBA) by employing dynamic phenylborate bonding [190]. The resultant NZ@hydrogel displayed notable characteristics including excellent injectability, self-healing properties, adhesion, and effective scavenging of free radicals. The hydrogel released metformin through interactions with CuPDA NPs, boric groups (B–N coordination), and the constrained structure of the hydrogel network. In addition to pH, glucose sensitivity, and photothermal responsiveness, it acted as an intelligent hydrogel platform to tackle bacterial infections and safeguard wounds from prolonged infection by sustained Cu^2+^ release. This hydrogel effectively facilitated cell recruitment and stimulated vascularization by releasing Cu^2+^, and decreased inflammation by removing ROS and simultaneously suppressing NF-κB signaling. Animal experiments validated that this combined action led to improved diabetic wound healing in rats by addressing both bacterial infections and inflammation, while promoting angiogenesis and deposition of ECM proteins compared to hydrogel groups without incorporated NZs.

The immune system plays a vital role throughout the entire wound healing process, which actively contributes to restoring equilibrium in damaged tissues through various mechanisms [191, 192]. Many studies have highlighted that the primary cause of persistent inflammation in skin defects is the disrupted transition of macrophages to the M2 phenotype [193, 194]. Tian et al. developed one such hydrogel dressing with antibacterial and inflammation modulation capabilities using a combination of bimetallic MOF-loaded with GO_*x*_ and a polyacrylamide (PAM)-based hydrogel. MOF (Fe–Cu) was made by hydrothermal method and endowed with POD-like properties. Subsequently, GO_*x*_ was applied to the MOF (Fe–Cu) via physical adsorption, establishing a self-activated cascade reaction system. The resultant MOF (Fe–Cu)/GO_*x*_ was polymerized with acrylamide (AM) and Bis-AM to produce hydrogel named as MOF(Fe–Cu)/GO_*x*_-PAM gel [168]. The MOF (Fe–Cu)/GO_*x*_-PAM gel facilitated the transition of macrophages into the M2 phenotype. Moreover, the anti-inflammatory environment induced by the hydrogel supports various stages of wound healing and aids in the efficient proliferation of new cells that produce collagen, which is essential for ECM formation. Additionally, the hydrogel facilitated the re-epithelialization process, by which the wound surface is covered with new epithelial cells to restore the protective skin barrier, which was not observed with pristine hydrogel. This transformation accelerated the transition of the wound microenvironment to a remodeled state that subsequently promoted angiogenesis and neurogenesis, which further aided in the wound healing process.

#### NZ@hydrogels for Photothermal Therapy

In recent times, a multitude of innovative therapeutic approaches have surfaced and among them, PTT has garnered significant interest [195]. This noninvasive treatment can address inflammatory diseases, particularly those caused by multidrug-resistant (MDR) bacterial infections [196, 197]. These hydrogels efficiently convert light energy into heat upon NIR irradiations and generate localized hyperthermia. This controlled release of heat not only attributes antibacterial effects but also stimulates various regenerative processes in the skin [198]. The photothermal effects induced by the hydrogels promote angiogenesis and improve nutrient and O_2_ supply to the regenerating tissue. Additionally, the heat generated by the NZs triggers collagen synthesis that leads to the production of collagen fibers, and the restoration of skin integrity and elasticity [199]. Given these multiple functions through PTT, the development of NZ@hydrogels with photothermal properties may be effective for handling bacteria-infected skin wounds.

For example, Wang et al. utilized carrageenan-based hydrogel incorporated with polyethylene glycol dicarboxylic acid (COOH–PEG–COOH)-modified zirconium-ferrocene MOF NSs (PEG@Zr-Fc MOF hydrogel) by COOH–PEG–COOH functionalization and physical assembly [169]. This hydrogel demonstrated the capability to capture both gram-positive and negative bacteria by exerting ROS destruction. Importantly, by incorporating the photothermal property, the enzymatic activity of the PEG@Zr-Fc MOF hydrogel was enhanced, leading to synergistic effects. In vivo experiments using bacteria infected wound model confirmed the potent bactericidal activity of the hydrogel. Compared to the pristine hydrogel group, the PEG@Zr-Fc MOF hydrogel demonstrated a more pronounced restoration of normal epithelial tissue and a denser arrangement of collagen fibers, indicating the successful re-epithelialization of the wound surface. Additionally, wounds subjected to the NZ@hydrogel treatment exhibited a diminished presence of inflammatory cells, indicating a more effective wound healing outcome.

Similarly, Li et al. considered a hydrogel called CTCS by combining Cu co-coordinated donor–acceptor type (D–A) covalent organic frameworks (COF)-like nanomaterial and SA [200]. This unique hydrogel exhibited both photocatalytic and anti-inflammatory properties effective for dealing with wound infections. The COF-like material within the CTCS hydrogel was activated by 660 nm light. The fundamental mechanism involves the establishment of a D–A COF-like structure with a π-conjugated system, where Cur functions as the electron donor and TCPP acts as the electron acceptor. Consequently, when exposed to 660 nm light, the hydrogel could eliminate bacteria within a remarkably short time attributed to the synergetic interactions of photocatalytic and photothermal processes. Furthermore, in vivo experiments demonstrated that the CTCS hydrogel not only reduced the expression of pro-inflammatory cytokines but also enhanced the expression of essential cytokines to promote wound healing.

Recently, Li et al. developed NZ@hydrogel with excellent photothermal activity by functionalizing MoS_2_ NSs with TA/Fe complex formed between TA and Fe [50]. The resultant MoS_2_@TA/Fe NSs were encapsulated in a hydrogel formed between PVA, Dex, and borax through the dynamic boron ester bonds. The hydrogel exhibited adhesive, self-healing, and shape-adaptable characteristics, aiming to accelerate the wound healing process. The NZ@hydrogel exhibited exceptional antibacterial efficacy through PTT, GSH depletion, and POD-mimic behavior in acidic conditions. Additionally, under neutral conditions, MoS_2_@TA/Fe-incorporated PVA/Dex hydrogel demonstrated CAT-mimic nature to deliver O_2_, antioxidant properties, and targeted reduction of inflammation through TA. The in vivo experiments in infected skin wounds confirmed that the NZ@hydrogel holds great potential for tackling bacteria-infected wound healing. By concurrently addressing infection, supplying O_2_, neutralizing free radicals, and mitigating inflammation, the PVA/Dex/MoS_2_@TA/Fe hydrogel establishes an optimal microenvironment conducive to cell proliferation, vascularization, granulation tissue formation, and re-epithelialization, leading to notable accelerated wound healing as compared to the pristine hydrogel.

In another study, Zhu et al. proposed an innovative strategy known as Iron-actuated Janus Ion Therapy (IJIT) to modulate iron metabolism in bacterial biofilms and immune cells [161]. The IJIT approach involves the development of a biofilm microenvironment (BME)-receptive photothermal microneedle (MN) patch named FGO@MN. This patch is created by growing Fe_3_O_4_ NPs on graphene oxide (GO) NSs (Fe_3_O_4_@GO), which are then incorporated within MN tips made of methacrylated HA (MeHA). Within the BME, the catalytic product of OH^•^ generated by FGO@MN impairs bacterial heat-shock proteins that sensitize the biofilm to thermal effects. Additionally, slight photothermal treatment activates the uptake of Fe leads to intracellular Fe overload, and induces ferroptosis-like death in the bacteria. Furthermore, the Fe-nourished neutrophils surrounding the BME can be rejuvenated thus restoring their suppressed antibiofilm function. This revival was evident through increased phagocytosis, chemotaxis, and inhibition of neutrophil extracellular trap formation, ultimately combining the elimination of bacterial biofilm infections. By combining the interference caused by heat stress-induced Fe regulation and the reactivation of Fe-nourished immune cells, an impressive elimination rate for bacterial biofilm infection could be achieved. Animal experiments further validated the successful eradication of bacteria within a few days, which underscores the promising potential of IJIT for future clinical applications, particularly in the treatment of bacterial biofilm infections associated with skin wounds.

#### NZ@hydrogels for Chemo-dynamic Therapy

CDT has originated as a propitious strategy for skin regeneration utilizing chemical reactions to generate ROS within the targeted microenvironment of the tissues [201]. In this approach, NZ@hydrogels function as catalysts and interact with naturally present H_2_O_2_ in the tissue usually in a mildly acidic environment that triggers the generation of extremely cytotoxic ROS, notably OH^•^ radicals, through Fenton or Fenton-like reactions [202]. The ROS generated facilitates angiogenesis, tissue nourishment, and oxygenation. Additionally, ROS stimulates the production of growth factors, ECM components, and signaling molecules involved in tissue remodeling [203]. Furthermore, controlled production of ROS through CDT can regulate inflammation, aiding in the transition from the inflammatory to the proliferative phase of tissue healing [8]. CDT, compared to other therapeutic strategies, enables localized ROS production within the tissue, thus minimizing potential harm to healthy surrounding tissues [204]. This technique can also be customized for specific tissue types and conditions by selecting appropriate NZs and adjusting the reaction conditions. Furthermore, the absence of external energy sources like light or heat simplifies the treatment process and allows application even into deep tissues. For these reasons, CDT has been used for skin regeneration [205, 206].

For instance, Zheng et al. introduced an adaptable thermo-gel named CuO_2_–BSO@Gel to tackle hypoxia connected to melanoma and bacterial infection associated with skin wounds simultaneously [207]. This NZ@hydrogel is composed of thermosensitive chitosan hydrogel, CuO_2_ NZ, and glutathione inhibitor l-Buthionine-(S, R)-sulfoximine (BSO) which possess dual functionality as sonosensitizers and chemo-dynamic agents. The unique characteristics of CuO_2_ NZs including a narrow bandgap and the presence of Cu^2+^ ions make them effective for both sonodynamic therapy (SDT) and CDT, respectively. Moreover, the NZs could self-generate H_2_O_2_, thereby enhancing the production of OH^•^ radicals through a Fenton-like reaction within the mildly acidic melanoma microenvironment and infected skin wound. Combined therapy, along with BSO, induces enhanced intracellular oxidative stress, leading to deactivation of glutathione-dependent peroxidase 4 (GPX4) and triggering ferroptosis in tumor cells. As a result, the CuO_2_–BSO@Gel serves as a temporary skin, providing a conducive, moist, and breathable environment while maintaining high drug concentrations to inhibit skin tumors and combat bacterial infections. Moreover, the hybrid chitosan hydrogel significantly accelerated healing in infected wounds by supporting effective chemo-sonodynamic antibacterial activity and angiogenesis. Consequently, this multifunctional NZ@hydrogel, characterized by injectability, adhesiveness, thermosensitivity, and antitumor and antibacterial properties, has shown promising in vivo results, demonstrating tumor growth inhibition efficiency, antibacterial and infected wound healing efficiency in Balb/c mice. Following healing, compared to pristine hydrogel, the CuO_2_–BSO@Gel group displayed fewer inflammatory cells, a continuous connection between the epidermis and dermis, higher collagen deposition, and an increased number of newly formed capillaries. Overall, the developed NZ@hydrogel was proposed as a wound dressing for addressing skin tumors, both suppressing tumor growth and facilitating simultaneous skin tissue healing.

In another study, injectable NZ@hydrogels were developed with strong tissue adhesion by incorporating Ag-doped molybdenum carbide (Mo_2_C) derived polyoxometalates (POM), urea (U), gelatin (G), and tea polyphenols (G) [208]. The primary aim was to achieve a synergistic effect, ensuring consistency, prolonged efficacy, and facilitation of the wound-healing process. Upon injection, the prepolymers formed an adhesive hydrogel in situ as U diffused. Ag-POM served as a pH-responsive photothermal agent, undergoing protonation and aggregating into larger nanoclusters with stronger absorption in NIR-II compared to POM, resulting in the exceptional photothermal performance of the resulting NZ@hydrogel. Moreover, the reaction between Mo^5+^ in Ag-POM and excess H_2_O_2_ in the inflammatory microenvironment generates toxic ^1^O_2_, effectively eradicating bacteria by CDT. The tissue adhesive property of the NZ@hydrogel ensured its safe attachment to the wound, guaranteeing consistent and continuous therapy. Simultaneously, this adhesion accelerates the wound-healing process. Both in vitro and in vivo experiments demonstrated that the T-G-U hydrogel could eliminate drug-resistant bacteria through microenvironment-responsive PTT and sustained ^1^O_2_ generation. Importantly, the Ag-POM/T-G-U hydrogel exhibited the highest wound closure rate after 3 days of PTT, surpassing 50%, which significantly outperformed the other experimental groups. This can be attributed to the rapid eradication of MRSA infection by NZ@hydrogel in the wound and their capacity to form a protective layer over the wound.

In a similar work, Dong et al. formulated Cu_2_O/Pt hydrogel by combining Cu_2_O/Pt nanocubes with a mixture of alginate (ALG) and HA by a simple assembly technique resulting in ALG-HA hydrogel [171]. The Cu_2_O/Pt hydrogel, in conjunction with GO_*x*_, offers an improved treatment method that targets both gram-positive and gram-negative bacteria using CDT. The GO_*x*_ enzyme in the hydrogel converts glucose to gluconic acid and H_2_O_2_, depriving bacteria of nutrients and leading to its death. Moreover, the Cu_2_O/Pt NZs released in an acidic environment react with H_2_O_2_ and generate toxic OH^•^ radicals for CDT. Additionally, gluconic acid lowers the pH, and Cu ions are released from the Cu_2_O/Pt hydrogel, which further enhances VEGF expression and promotes endothelial cell proliferation, migration, and angiogenesis for healing diabetic skin wounds. In vivo experiments on diabetic rats with *S. aureus* infection validated the accelerated rate of wound healing in those treated with Cu_2_O/Pt hydrogel compared with the control group. Furthermore, the administration of the Cu_2_O/Pt hydrogel + GO_*x*_ + NIR led to enhanced epithelial thickness and regeneration of hair follicles and mature sebaceous glands.

Recently, Wang et al. developed a NZ@hydrogel called PHF by incorporating a hollow-structured Cu_2_MoS_4_ NZ (HCMS) modified with polyethyleneimine-vancomycin (PV) copolymers (PV@HCMS) into a thermosensitive poloxamer F127 hydrogel [172]. The PHF hydrogel possessed remarkable skin regeneration properties along with good antimicrobial activity, making it an ideal solution for the synergetic treatment of burn wound infections. The NZ@hydrogel enabled synergistic therapy by combining NIR-II (1064 nm) PTT and CDT, exhibiting exceptional catalytic performance similar to PODs, enabling the generation of highly detrimental OH^•^ through NIR-II-enhanced Fenton-like reactions that impart antibacterial properties. The in vivo results demonstrated that compared to the control groups, PHF facilitated anti-inflammatory effects and re-epithelialization in burn skin wounds, indicating their potential for use as wound dressings.

In another study, Zhu et al. developed a smart NZ@hydrogel by integrating TMB/Fe^2+^/GO_*x*_ into thermosensitive Pluronic F-127 (PF127) hydrogels [209]. When the TMB/Fe^2+^/GO_*X*_/PF127 hydrogel was applied as a wound dressing for diabetic patients, it catalyzed the conversion of colorless TMB into oxidized TMB. The loaded GO_*x*_ degrades blood glucose producing H_2_O_2_ and gluconic acid to facilitate the Fe^2+^-based Fenton reaction which generates OH^•^ that assists TMB oxidization. A color change was also observed as TMB oxidation occurs which corresponds to the blood glucose levels ranging from 1 to 10 mM, which can be monitored using a smart phone. Simultaneously, this initiates CDT by producing OH^•^ specifically for bacterial elimination. Furthermore, the oxidized TMB exhibited strong absorption in the NIR range enabling PTT. This combined CDT/PTT resulted in bacterial elimination and the infected skin subsequently underwent complete repair, making TMB/Fe^2+^/GO_*X*_/PF127 a promising platform for the treatment of diabetic wounds.

Although still in infancy, many studies have shown promising results with NZ@hydrogels in the context of CDT. The continuous research in this field hence holds great potential for advancing CDT and further improving the outcomes of skin tissue regeneration. In clinical settings, there is an urgent need to develop intelligent designs for novel antibacterial approaches, aimed at diagnosing and treating chronic multidrug-resistant bacterial infections [210]. However, the challenge lies in achieving precise theranostics that target specific inflammatory microenvironments.

One recent study addressed this issue by introducing infection ambiance-activated core–shell Gd-doped Bi_2_S_3_@Cu(II) boron imidazolate framework (Bi_2_S_3_:Gd@Cu-BIF) nano-assemblies synthesized via solvent-assisted self-assembly [211]. Upon exposure to an 808 nm laser, the nano assemblies demonstrated exceptional photothermal conversion and proved advantageous for CDT. They generated ^1^O_2_ and OH^•^ by depleting intracellular glutathione and catalyzing the decay of endogenous H_2_O_2_ within the inflammatory microenvironment, effectively combating bacterial infections and accelerating wound healing. The Cu^+^/Cu^2+^ ions released from Cu-BIF participate in intracellular in situ Russell and Fenton-like catalytic reactions, generating ROS to disrupt bacterial redox balance in the inflammatory microenvironment. Notably, the nanoassemblies exhibited robust broad-spectrum antibacterial properties, achieving a 99% inhibition rate against E. coli and MRSA in vitro. Moreover, in vivo studies on wound healing revealed that Bi_2_S_3_:Gd@Cu-BIF nanoassemblies functioned effectively as a wound spray, expediting healing following MRSA infections through synergistic PTT/CDT. The wound healing rate in the synergistic treatment group reached 99.8%, surpassing the 69.5% rate in the control group. The synergistic PTT/CDT anti-infection strategy accelerates wound healing by supporting endothelial cell angiogenesis and fibroblast migration. Additionally, the nanoassemblies facilitated magnetic resonance and computed tomography dual-modal imaging, demonstrating promising potential as an integrated diagnostic nanoplatform.

#### NZ@hydrogels for Oxygen Generation

The O_2_ generation by NZ@hydrogels has also special implications for skin regeneration because O_2_ is involved in skin rejuvenation by supporting cell survival, proliferation, and tissue restoration [159, 212]. However, chronic wounds or infections can impede the O_2_ supply to damaged tissues, inhibiting the healing process. The O_2_-generating hydrogels are designed to address this challenge by providing a targeted and continuous release of O_2_ at the wound site. The NZ@hydrogels act as catalysts, breaking down endogenous or exogenous H_2_O_2_, leading to the production of O_2_ and H_2_O.

One recent study by Li et al. developed NZ@hydrogels as ROS-driven oxygenerators to enhance the healing of diabetic wounds. This hydrogel utilized hydrazide-adapted HA and aldehyde-modified HA (HA-HYD) along with a CAT-mimic NZ derived from mesoporous manganese cobalt oxide (MnCoO) coated with ε-polylysine (MnCoO@PLE/HA) [159]. The NZ@hydrogel demonstrated outstanding biocompatibility, injectability, and remarkable self-healing capacity. Furthermore, it effectively captured elevated levels of ROS in diabetic wounds while concurrently initiating ROS-driven O_2_ production. The NZ@hydrogel protected skin cells, including fibroblasts and endothelial cells, against ROS, thereby reducing hypoxia-induced cell death and proliferation inhibition. It also induced macrophage polarization toward the anti-inflammatory M2 phenotype. The in vivo results demonstrated that wounds treated with the MnCoO@PLE/HA hydrogels achieved approximately 41.5% closure by the 8th day, surpassing the 20.7% closure of the PBS group and 29.4% closure of the pristine hydrogel group. By the 16th day, wounds treated with the NZ@hydrogel had nearly fully healed, whereas the remaining wound areas in the PBS group and pristine hydrogel were 46.8% and 35.7%, respectively. These findings underscore the substantial acceleration of wound closure with the MnCoO@PLE/HA hydrogel dressing compared with the other groups. Additionally, the regenerated tissues in the MnCoO@Gel Group exhibited a clean wound microenvironment, superior granulation tissue formation, and rapid morphological healing compared to the other two groups, highlighting the effectiveness of these NZ@hydrogels in promoting wound healing under diabetic conditions.

Recently, Shang et al. developed a multienzymatic NZ@hydrogel spray called ACPCAH, comprising ultrasound-responsive HA-encapsulated l-arginine, ultrasmall Au, and Cu_1.6_O co-loaded phosphorus-doped graphitic carbon nitride NSs [88]. The rheological analysis of ACPCAH hydrogel revealed a consistently lower and stable storage modulus compared to its loss modulus, indicating maintained viscosity suitable for a sprayable hydrogel. Both ACPCAH and HA hydrogels exhibited sustained high viscosity at low shear rates, transitioning to low viscosity at high shear rates, suggesting that the addition of Au/Cu_1.6_O/P–C_3_N_5_ NZs did not alter the mechanical properties of hydrogels. Moreover, ACPCAH displayed multifunctional properties, including O_2_ generation capacity, and anti-inflammatory and antibacterial properties for treating diabetic wounds. The encapsulation of HA enhanced the biocompatibility and stability of NZs, allowing targeted decomposition by hyaluronidase in bacterial biofilms which released l-arginine and NZs to strengthen bacterial interactions. ACPCAH hydrogels can be activated by the diabetic wound microenvironment to initiate a cascade reaction that mimics the activity of SOD, CAT, POD, GO_*x*_, and NOS. This therapeutic spray was well-adapted to diabetic wounds and promoted regeneration in vivo by reducing blood glucose levels, and reducing inflammation and hypoxia while supporting blood vessel formation.

In another study, Zhu et al. developed a hydrogel with multiple enzyme-like activities using CDs/AgNPs and Cu, Fe-NC [119]. During the inflammatory stage of wound healing, the system functioned as a CAT mimic, generating sufficient O_2_ by catalyzing intracellular H_2_O_2_ to alleviate hypoxia. The capacity of NZ@hydrogel to reduce GSH and perform OXD-like functions by breaking down O_2_ to generate O_2_^•−^ and OH**˙** demonstrated outstanding antibacterial efficacy. The presence of catechol groups on the CDs/AgNPs imparted adhesive properties to the hydrogel, resembling those found in mussels, attributed to their dynamic redox equilibrium properties. This NZ@hydrogel proves highly effective in promoting wound healing by addressing bacterial infections and optimizing the performance of NZs.

Similarly, Wang et al. also developed a NZ@hydrogel named EGAP@HG by incorporating CAT-mimic NPs (EGAP) into a thermosensitive hydrogel [163]. The thermoresponsive hydrogels were made by combining amphiphilic nonionic block polymers, specifically Poloxamer-188 into Poloxamer-407 with a critical gelation temperature of 37 °C, achieved at a Poloxamer-407 concentration of 17.9% and Poloxamer-188 concentration of 8%. The EGAP@HG demonstrated injectability and autonomous self-healing ability through repeated cycles. The adhesive strength of both the hydrogel and EGAP@HG also suggests their potential as effective hydrogel adhesives in wound healing. The EGAP@HG system establishes a protective physical barrier on the wound, fostering a moist environment. Initially, EGAP converts H_2_O_2_ to O_2_ at the wound site, alleviating local hypoxia. Subsequently, released EGF promotes the proliferation of epidermal cells. Besides, gallic acid from the outer EGAP layer exhibits anti-inflammatory and antioxidant effects. Finally, Ag^+^ ions from inner templates (APs) have bactericidal properties, effectively eliminating bacteria. This offered several advantages for diabetic wound management by improving the inflammatory microenvironment.

Li et al. also developed a defect-rich MoS_2_@Au@BSA NZs for enhancing diabetic wound healing when integrated with an injectable hydrogel [133]. The hydrogel, formed by cross-linking ODex and gC through Schiff base chemistry, exhibits notable tissue adhesion. This covalent Schiff base network enabled the creation of a protective barrier, preventing bacterial invasion and maintaining a conducive moist environment for optimal wound healing. Additionally, ODex/gC/MoS_2_@Au@BSA demonstrated injectability and shape adaptability. The self-healing and rheological tests further confirmed the self-recovery property of ODex/gC/MoS_2_@Au@BSA NZ@hydrogels. Due to their small size, the hybrid NZs showed an improved GO_*x*_-like activity. Particularly, AuNPs facilitated the oxidation of glucose, which generates gluconic acid and H_2_O_2_, which were further converted into OH**˙** through the POD-mimic nature of the MoS_2_@Au@BSA NSs within the acidic wound microenvironment. Consequently, the cascade reaction of the NZs effectively consumed excessive glucose and eradicated the bacteria, thereby accelerating the healing process. As the diabetic wound progresses to the alkalescent stage, the hydrogel exhibits CAT and SOD-like properties that result in abundant production of O_2_ and efficient scavenging of ROS. The generated O_2_ then alleviated hypoxia and facilitated glucose oxidation, ensuring a continuous and normal cascade reaction to aid the wound healing process by promoting collagen deposition, angiogenesis, and epithelialization.

## Therapeutic Efficacy of NZ@Hydrogels in Clinically Relevant Diseased and Wounded Skin Tissues

We have witnessed that the NZ@hydrogels with different combinations have been developed with a diverse set of key therapeutic functions, including antioxidative, antibacterial, and anti-inflammatory activities. This part discusses the cutting-edge systems that have demonstrated in vivo efficacy in clinically relevant conditions related to skin wounds and diseases, especially challenging cases such as diabetic wounds, infected wounds, atopic dermatitis, and radiation-induced skin injuries (RISI).

Diabetic wound healing, known for its complexities in patients with diabetes, greatly benefits from the application of NZ@hydrogels. These materials exhibit remarkable antioxidative abilities that mitigate the detrimental effects of excessive ROS at the wound site thus accelerating tissue regeneration and reducing inflammation. Similarly, NZ@hydrogels showed exceptional antimicrobial properties for the treatment of infected wounds. Their catalytic activity facilitates the rapid breakdown of bacterial biofilms and effectively eliminates infections thereby fostering wound healing. Moreover, in cases of dermatitis, NZ@hydrogels alleviate symptoms by neutralizing inflammatory mediators, soothing irritation, and restoring the protective barrier function of the skin. Furthermore, these hydrogels play a vital role in addressing RISI by neutralizing ROS and promoting tissue repair mechanisms, ultimately enhancing recovery, and minimizing long-term side effects.

### Diabetic Skin Wound Healing

Cutaneous wounds in diabetic patients are becoming a significant threat to the global healthcare system [213]. Diabetic skin wound healing poses a clinical challenge as this disease is characterized by complex pathological complications such as hypoxia, and chronic inflammation [214]. These conditions hinder angiogenesis, making the healing process more difficult due to inadequate nutrient supply. Additionally, this microenvironment promotes bacterial colonization, compromises immune system function, and leads to elevated oxidative stress which is harmful to healthy cells. Despite the presence of standardized treatment methods in clinical practice, the process of effectively healing diabetic wounds continues to face substantial challenges.

**Fig. 5:**
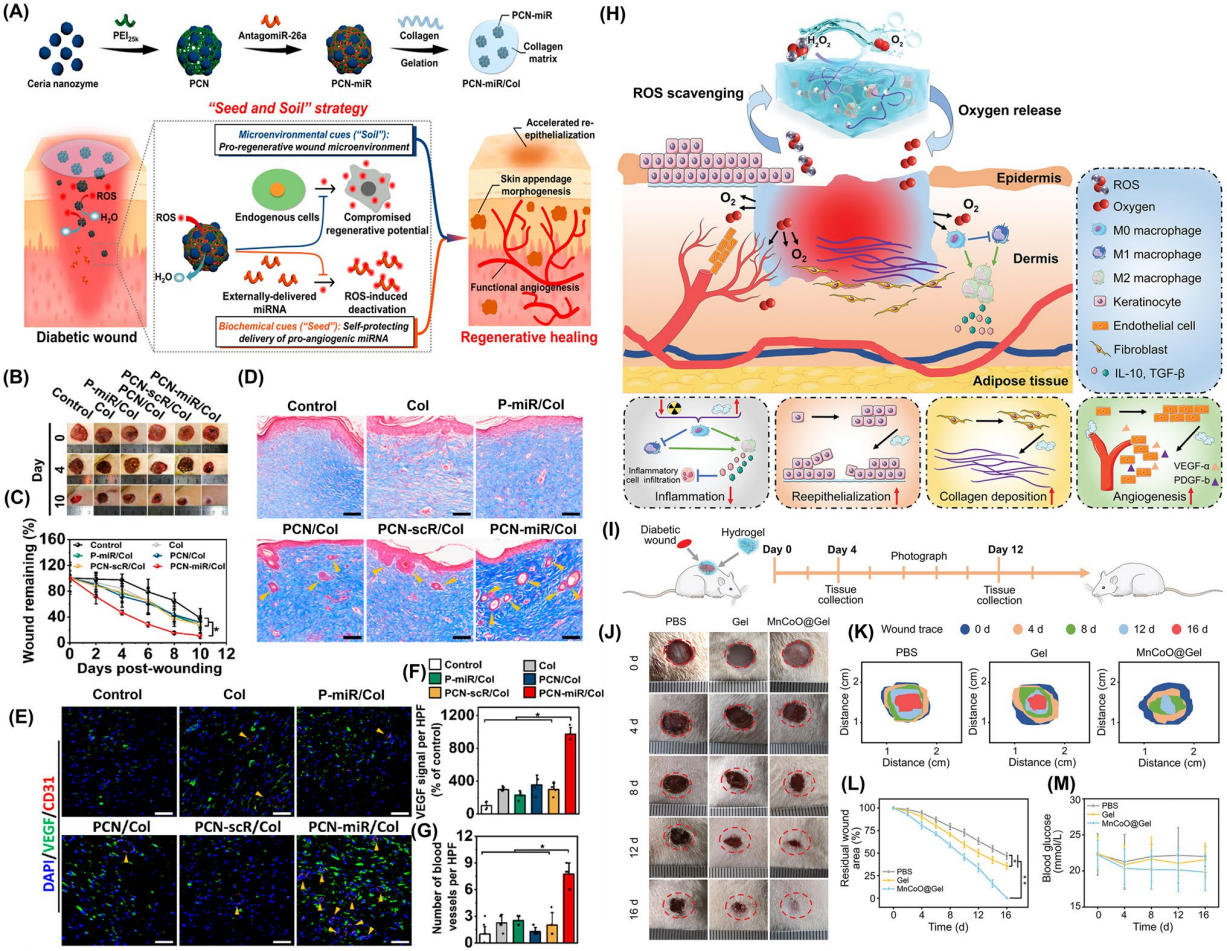
Diabetic wound healing accelerated by NZ@hydrogels. **a** An illustration showing the fabrication process of PCN-miR/Col hydrogel for hastening diabetic skin regeneration. **b** Photographs of diabetic wounds at different periods after the hydrogel treatment. **b** Quantification of wound area obtained from photographs. **d** Masson’s trichrome staining of skin tissues after 28 days of treatment. Scale bars, 100 μm. **e** Fluorescent expression of VEGF and CD31 markers. Scale bars, 50 μm. **f** Quantification for VEGF expression and **g** Quantification of several blood vessels at 28 days [45]. Copyright 2019, American Chemical Society. **h** Representation of the accelerated wound healing coordinated by MnCoO@PLE/HA hydrogel. **i** An illustration showing the fabrication process of MnCoO@PLE/HA hydrogels for diabetic wound healing. **j** Photographs of diabetic wounds were evaluated up to 16 days after the hydrogel treatment. **k** A representation of relative wound healing at different time points. **l** Quantification of residual wound area from wound photographs. **m** Blood glucose levels in animals induced with diabetes for studying the wound healing process [159]. Copyright 2022, John Wiley and Sons, Inc

Among the numerous strategies aimed at tackling the difficulties linked to diabetic wound healing, one particular concern revolves around the functional restoration of blood vessels [215]. A recent study by Wu et al. proposed to repair the oxidative wound microenvironment into a pro-regenerative state while delivering proangiogenic miRNA cues [45]. This was achieved using a miRNA-loaded, redox-regulatory ceria NZ (CN)-encapsulated self-protecting hydrogel (PCN-miR/Col). Collagen, an inherent component of the ECM, was utilized in the fabrication of the hydrogel, providing an advantageous substrate for the incorporation of PCN-miR. To counteract the effects of the antiangiogenic miR-26a, a known target exacerbated by hyperglycemia and implicated in impaired angiogenesis in diabetic wounds, AntagomiR-26a was employed for inhibition. This approach aims to address the specific challenges associated with diabetic wound healing. The PCN-miR/Col hydrogel not only reshaped the unreceptive oxidative wound microenvironment but also ensured the structural integrity of the incorporated proangiogenic miRNA within these oxidative settings (Fig. 5a). Diabetic wounds treated with these PCN-miR/Col therapeutic hydrogels exhibited significantly accelerated wound closure and improved wound quality which are characterized by highly organized alignment of collagen fibers, morphological development of skin appendages, enhanced growth of new blood vessels and improved O_2_ saturation in the healed wound (Fig. 5b–g). The PCN-miR/Col treatment group demonstrated significantly accelerated wound closure compared with the other groups. SEM imaging revealed well-ordered collagen fiber alignment in the treated wounds, in contrast to the disarranged fibers in the control groups. Picro Sirius red staining confirmed increased collagen deposition in PCN-miR/Col-treated wounds, in contrast to other groups. The treatment also led to a significantly increased type I/III collagen ratio, indicating successful collagen maturation and enhanced mechanical strength. Diabetic wounds treated with PCN-miR/Col also showed pronounced VEGF expression and increased blood vessel formation.

Among various alternatives, hydrogels functionalized with NZs have garnered considerable attention as potential wound dressings for addressing diabetic skin wounds [214]. For instance, Li et al. developed a hydrogel inspired by biological metabolism, which is specifically designed to improve the unfavorable microenvironment found in diabetic wounds [159]. The NZ@hydrogel consisted of well-dispersed MnCoO NZ made using MOF as templates. To ensure both biocompatibility and functionality, a surface modification of MnCoO NZ was conducted by coating them with ε-polylysine (EPL), which is rich in –NH_2_ groups. This coating process involved electrostatic interactions, resulting in well-dispersed MnCoO@PLE NZs. The hydrogel part consisted of HA, a prominent polysaccharide known for its excellent biocompatibility, biodegradability, and abundant presence in nature. Due to its remarkable bio-functionality, it also plays a crucial role in preserving moisture and effectively safeguarding the skin. Here the NZ serves a dual function within the polymeric hydrogel network, serving as both a cross-linker through dynamic chemical bonds and a catalytic site. The MnCoO@PLE/HA hydrogel not only captured excessive ROS in diabetic wounds but also generated O_2_ through a ROS-aided O_2_ production mechanism (Fig. 5h). Consequently, this hydrogel protected dermal cells from ROS and hypoxia-induced cell damage and inhibition of cell proliferation. When applied to diabetic wounds, significant improvements were observed in the expression of M2 phenotypes, cell growth, re-epithelialization, collagen deposition, and neovascularization (Fig. 5i–m) compared to control groups. MnCoO@PLE/HA hydrogel-treated wounds showed remarkable progress, achieving approximately 41.5% closure by the 8th day, surpassing the 20.7% of the PBS group and 29.4% of the pristine hydrogel group. By the 16th day, MnCoO@PLE/HA hydrogel-treated wounds almost completely healed, with residual areas of 46.8% in the PBS group and 35.7% in the pristine group. Pristine hydrogel also contributed to accelerating wound healing compared to the PBS group, likely due to its inherent benefits in maintaining a moist healing environment and acting as a bacterial barrier. Additionally, blood glucose levels in animals treated with MnCoO@Gel exhibited a slight decrease over time, potentially associated with antioxidant therapy for diabetes.

The exploration of NZ@hydrogels and their application in medical imaging for real-time blood glucose level detection is an area yet to be explored. The integration of "diagnosis and treatment" functions through precision medicine in diabetic wound repair holds significant potential [216]. For this purpose, Han et al. developed a self-healing injectable HA hydrogel using a Schiff base reaction [160]. GO_*x*_–MnO_2_ NZs were synthesized and VEGF-loaded nanobubbles were created and subsequently loaded into the hydrogels. The US@GO_*x*_@VEGF hydrogel enabled real-time monitoring of glucose concentration while delivering VEGF precisely and noninvasively through ultrasound stimulation to deplete glucose levels. Additionally, this hydrogel could monitor glucose levels by magnetic resonance imaging (MRI). Hence, the system enabled real-time monitoring of glucose levels while maintaining its homeostasis through noninvasive interventions as well as accelerated the healing process of diabetic skin defects, which demonstrates its diagnostic and therapeutic functions.

As witnessed, most studies have focused on removing ROS without effectively regulating the continual and excessive generation of ROS by controlling inflammation levels. Disrupting the interrelated inflammatory phenomena has been proposed by concurrently hunting ROS and detaining pro-inflammatory factors [217]. This approach can safeguard against the detrimental effects of unwarranted ROS production both downstream and upstream, ultimately hastening diabetic wound regeneration. One recent study presented a hydrogel termed Cu_5.4_O@Hep-PEG for the simultaneous suppression of persistent proinflammatory chemokines and mitigation of high oxidative stress in inflammatory diseases [170]. The hydrogel effectively scavenged pro-inflammatory proteins such as monocyte chemoattractant protein-1 (MCP-1) and interleukin-8 (IL-8) in wound fluids and thereby reduced the migratory activity of polymorphonuclear neutrophils and monocytes. Moreover, this therapeutic hydrogel exhibited multienzymatic activity and protects the wound tissue from ROS-related damage. The goal of this approach was not to completely suppress inflammation but rather to maintain a balanced response for wound healing while minimizing unwanted side effects.

The MOF-based nanomaterials combine metal nodes and organic ligands in customizable ways and thus have gained significant attention as a therapeutic material for skin regeneration [218]. Chao et al. utilized MOF-818 and its SOD and CAT-mimic properties for antioxidant defense in diabetic skin regeneration [75]. They combined MOF NZs with copolymers comprised of poly(lactic acid-*co*-glycolic acid) and poly(ethylene glycol) which forms thermosensitive gel (PLGA-PEG-PLGA), resulting in an antioxidative system called MOF/gel that facilitates the healing of chronic wounds in diabetic rats. The gel effectively retained the MOF NZs at the wound sites, thus ensuring a sustained treatment effect by modulating the oxidative microenvironment. Impressively, a single dose of MOF/gel initially demonstrated efficacy comparable to that of a daily applied clinical drug (HEGFG) and the non-diabetic group, outperforming the gel and nCe/gel controls. By day 12, both the MOF/gel and HEGFG groups achieved complete re-epithelialization compared to the pristine Gel group. Further analysis revealed increased blood vessel density, more collagen fibers, and elevated growth factor expression in the MOF/gel and HEGFG groups than in the gel group. The MOF/gel-treated wounds also exhibited higher levels of M2 polarized macrophages. Further analysis showed reduced pro-inflammatory cytokine expression, especially in NZ@hydrogels, highlighting their effectiveness in treating chronic diabetic wounds.

A different study by Deng et al. combined GO_*x*_ and quasi-amorphous Fe_2_O_3_ into Zn-MOF (F-GZ) to take advantage of the cascade enzyme catalytic activity and antibacterial effects for targeting the specific microenvironments found in diabetic wounds [164]. Subsequently, F-GZ was encapsulated inside an injectable and self-repairable hydrogel made of oxidized HA (OHA) and N-carboxyethyl chitosan (CEC) by copolymerization to form a nanogel (F-GZ@G). In addition to the enzyme-mimic and antibacterial effects attributed to the NZ@hydrogel, this study also observed an interesting phenomenon of general innate immunity activation in diabetic wounds infected with bacteria. However, as the multifunctional F-GZ@G hydrogel was administered, the immune activation gradually decreased. Notably, the hydrogel specifically inhibited immune activation at pancreatic islet sites which leads to the recovery of impaired islets as evidenced by the enhance in glucose tolerance and improved insulin secretion. The ability of the hydrogel to suppress immune activation at pancreatic islets was linked to the replenishment of Zn^2+^ from the multifunctional hydrogel that results in reduced expression of zinc transporter 8 (ZnT_8_, antigens). Hence, this study presents an innovative approach to tackle diabetic wounds with the potential to impact diabetic immunity.

### Bacteria-Infected Skin Wound Healing

The prevalence of wound infections poses significant economic and social burdens on public healthcare systems [219, 220]. Considerable efforts have been devoted to the expansion of antibiotics, antibacterial nanomaterials or peptides, and cationic polymers to tackle the issue of bacterial infections [221]. While these strategies have demonstrated potential in inhibiting bacterial growth and facilitating skin wound healing, it is essential to acknowledge that these antimicrobial substances not only destroy harmful bacteria but also impede the growth of beneficial bacteria that disturb the equilibrium of microbial ecosystems in the skin. As such, there is an immense need to explore alternative approaches capable of curbing the proliferation of harmful bacteria while preserving the growth of beneficial bacteria [222–224]. Moreover, combating multidrug-resistant bacterial infections is a challenging task [225, 226].

To address these issues, NZ@hydrogels have been utilized to catalyze the generation of various ROS thereby destroying bacteria causing wound infections [128, 165]. In a recent study, CNT-loaded MoS_2_ NSs (CNT@MoS_2_) with triple enzymatic activities were combined with multifunctional hydrogels to effectively eliminate bacteria while neutralizing free radicals [83]. The hydrogel demonstrated remarkable antibacterial activity that was primarily attributed to its POD-mimic nature. It also facilitated GSH loss and employed PTT to further enhance the antibacterial effects. Of note, the NZ@hydrogel exhibited multienzymatic activity by converting O_2_^•−^ into H_2_O_2_ and O_2_ and then converting H_2_O_2_ into O_2_. Ultimately, the hydrogel could demonstrate excellent free radical scavenging capability in a neutral environment while providing an abundant supply of O_2_ for wound healing. Besides, the multifunctional hydrogel cross-linked through dynamic boron ester bonds unveiled adhesiveness, self-repairability, and shape-adaptive properties that effectively benefit the filling of irregular wound cavities.

MoS_2_-based NZs are multifunctional nanomaterials that combine intrinsic POD-like activity with high photothermal conversion efficiency in the NIR region [227]. NIR is known for its noninvasive treatment capability and has found applications in various fields, particularly in antibacterial therapy [228]. Building on this, Sang et al. developed a positively charged and multiporous NZ-hydrogel by incorporating MoS_2_ nanoflowers into a hydrogel composed of pNIPAAM (*N*-isopropylacrylamide)-AAM (acrylamide)-DMPA (*N*-[3-(dimethylamino)propyl] methacrylamide) [165]. The MoS_2_-hydrogel effectively captured and confined bacteria within the range of ROS damage through electrostatic interactions by significantly enhancing the antimicrobial efficiency of OH^•^ radicals. Furthermore, when exposed to 808 nm laser irradiation it resulted in synergistic antibacterial effects. Remarkably, the removal of the hydrogel-containing dead bacteria led to a notable reduction in inflammation occurrence that subsequently resulted in an accelerated progression of dermal wound healing.

Similarly, Ag NZs were also applied for this purpose by integration with bioinspired double-layer hydrogels [56]. To prepare double-layer hydrogels, carboxymethyl chitosan, and PVA were dissolved independently, and PEG was subsequently added. After casting and freezing, an upper hydrogel layer was formed. A solution of PVA, SA, and PEG was then poured onto the surface and frozen, resulting in the formation of a double-layer hydrogel. In the bi-layered hydrogel, the one with a large pore absorbs wound exudate and facilitates O_2_ exchange, and the other with small pores maintains a moist environment that prevents bacterial intrusion. By PDA coating to decrease the size of Ag NZs, the hydrogel demonstrated strong absorption of NIR light at 808 nm that generates heat and augments the production of OH^•^ radicals thus mimicking the action of PODs. These characteristics confer exceptional antibacterial properties when combined with the release of Ag^+^ ions. Additionally, the hydrogel exhibits adhesiveness due to the presence of catechol groups on the PDA molecules. Animal experiments have validated that the bilayer NZ@hydrogel expedited the generation of new skin in infected wounds by increasing collagen deposition and VEGF expression while diminishing TNF-α secretion as compared to the pristine hydrogels.

**Fig. 6:**
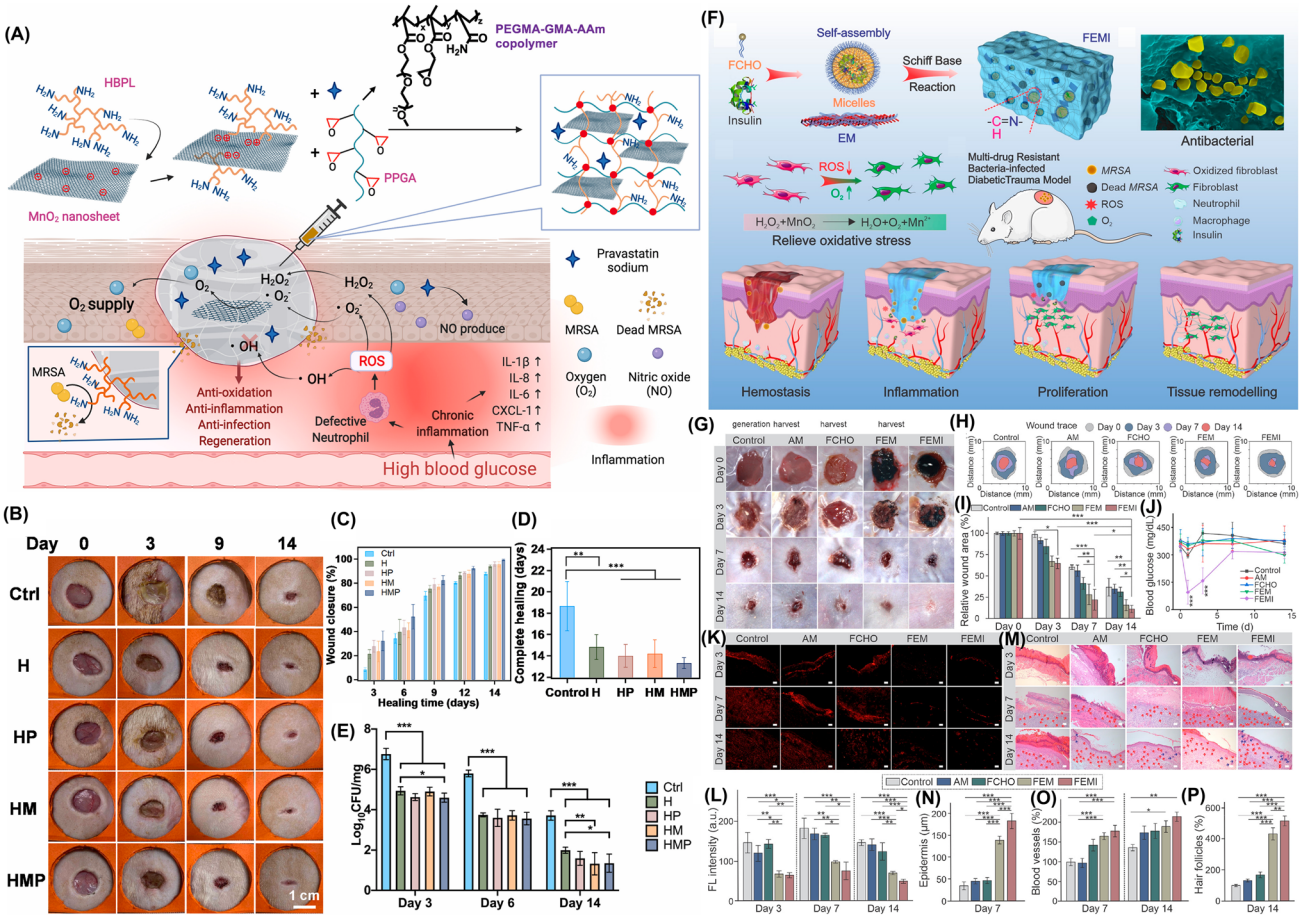
Wound healing process accelerated by NZ@hydrogels with antibacterial properties. **a** Fabrication of a multifunctional anti-bacterial hydrogel based on PEGMA-GMA-Aam copolymer. **b** Photographs of infected wounds after hydrogel treatment. Scale bar: 1 cm. **c** Relative wound healing rates at different time points. **d** Number of days taken for complete wound healing. **e** Quantification of bacterial density in the infected wound [158]. Copyright 2022, Elsevier. **f** An illustration of the fabrication of FEMI hydrogel for infected diabetic wounds. **g** Photographs of the wound after hydrogel administration. **h** A representation of the wound closure at different time points. **i** Quantification of wound closure rates. **j** Blood glucose levels were examined in mice at different time points. **k** The fluorescent expression of ROS levels in wounds by dihydroethidium (DHE). Scale bar: 50 μm. **l** The fluorescence intensity of DHE for various hydrogel groups. **m** Histological examination of wounds following hydrogel treatment. Scale bar: 100 μm. **n** Epidermal thickness examined on the 7th day. **o** Blood vessel regeneration examined on the 7th and 14th days. **p** Hair follicle formation examined on the 14th day [41]. Copyright 2020, American Chemical Society

Another study utilized a therapeutic hydrogel called HMP with multifunctional properties including antioxidant, anti-inflammatory, O_2_-generating, and antibacterial properties to combat bacterial infection-related inflammation (Fig. 6a) [158]. In animal experiments, this NZ@hydrogel exhibited an excellent capacity for treating bacteria-induced infection and accelerating wound closure during the inflammatory phase. In addition to alleviating detrimental ROS, the hydrogels reduced the inflammatory factors while simultaneously increasing the anti-inflammatory cytokines. Furthermore, the hydrogels stimulated TGF-β secretion, which aids neovascularization and collagen deposition that leads to effective skin regeneration (Fig. 6b–e). Rats treated with antibacterial hydrogels showed accelerated wound closure, notably the HMP hydrogel group with 32.2% closure on day 3, surpassing other groups. Upon discontinuation, the difference in closure among groups gradually reduced. All hydrogel-treated wounds healed faster than the control group, with the HMP group exhibiting the highest rate. The HMP group had the shortest healing time, suggesting superior promotion of infected diabetic skin wound healing through HMP hydrogel treatment.

Bacterial infections under diabetic conditions pose a significant challenge and are common complications that can exacerbate the complex nature of diabetes [229]. Owing to various factors associated with diabetes, the ability of the immune system to combat infections may be compromised which makes individuals more susceptible to bacterial infections. To address this issue, Wang et al. considered a hydrogel called “FEMI” by employing Schiff-based chemistry between ε-polylysine (EPL)-coated MnO_2_ NSs (EM) with insulin-laden self-assembled aldehyde Pluronic F127 (FCHO) micelles [41] (Fig. 6f). The hydrogel displayed injectability, self-healing, adhesive property and remarkable antibacterial properties against MDR bacteria through the synergistic combination of EPL and "nano knife-like" MnO_2_ NSs by breaking down endogenous H_2_O_2_ into O_2_. Additionally, this NZ@hydrogel was responsive to both pH and redox conditions that ensure the sustained and spatiotemporal precise release of insulin, which controls blood glucose levels. Animal experiments showed that hydrogels could hasten the healing rate of MDR bacteria-infected diabetic wounds, which demonstrates a possible effective strategy to combat bacterial infections in diabetic-diseased patients (Fig. 6g–p). FEMI hydrogels exhibited effective bactericidal action on day 3, surpassing other groups. FEMI hydrogels showed almost 100% reduction of bacterial colonies on day 14. Wounds treated with hydrogels exhibited significantly accelerated closure, with FEMI demonstrating the highest rate. Blood glucose and insulin levels were monitored, showing an increase in plasma insulin level and a subsequent decrease in blood glucose level by day 5. FEMI hydrogels exhibited excellent antioxidative performance indicated by lower ROS production, attributed to reduced glucose levels. Histo-morphological studies showed reduced inflammation and enhanced cell proliferation in the FEMI group. Additionally, wound re-epithelialization, granulation tissue formation, and angiogenesis were also significantly improved in the FEMI group compared to the pristine hydrogel group.

One of the challenging wound healing areas is the sites that undergo frequent motion such as joints and the neck as these areas hold a high risk of bacterial infection and inflammation [230]. One recent study by Li et al. addressed this issue by exploring a composite hydrogel (MPH) that consists of MoS_2_-PDA NZ and hydrogel copolymerized of *N*-isopropylacrylamide, acrylamide, and PF127-DA [166]. The MPH hydrogel possessed excellent mechanical properties and adhesiveness that enabled a quick adaptation to the movements of movable body parts. Interestingly, the hydrogel could closely conform to the wound thus preventing bacterial invasion and eliminating the need for additional fixation of dressings. Furthermore, the hydrogel exhibited photothermal properties with antibacterial effects along with intrinsic enzyme-like activity and antioxidant properties. This multifunctionality allowed the hydrogel to eliminate infections, reduce oxidative stress, and improve the wound microenvironment facilitating a faster healing rate in mice.

Among other forms of materials to address infected wounds, patches with intelligent properties have gained great interest for both the treatment and sensing of bacteria. One recent study reported smart colorimetric MN patches that combined with Fe ion-gallic acid coordination polymer nanodots (FNDs) to enable on-demand treatment and live monitoring of infected wounds [231]. The FNDs were synthesized through coordination reactions involving polyvinylpyrrolidone (PVP), gallic acid, and Fe ions and were filled into the MNs to form FNDs-MNs. The FNDs-MNs benefit from the pH-dependent POD-mimic nature of the FNDs, allowing for catalyzing H_2_O_2_ and producing a higher amount of OH specifically in acidic conditions that effectively destroys bacteria. Additionally, the FNDs-MNs exhibited color changes depending on the pH and H_2_O_2_ concentration, providing timely information on the wound healing progress and infection status. As the pH value decreased, the color of FNDs-MNs faded, suggesting that FNDs-MNs exhibit pH-dependent colorimetric properties. Additionally, when immersed, the FNDs-MNs displayed a time-dependent gradient shift in color corresponding to the increasing H_2_O_2_ concentration. Subsequently, an experimental setup was designed to replicate the microenvironment of MRSA-infected wounds using 100 μM H_2_O_2_ at pH 5.5. Over time, the color of FNDs-MNs gradually faded, whereas no distinct color variations were observed in the control solution of the same concentration at pH 7.4 in equal time intervals. Notably, FNDs-MNs exhibited no visible color changes when exposed to saline and cell culture media. Therefore, FNDs-MNs possess in vitro colorimetric properties, particularly within environments that mimic bacterial-infected wounds. When applied on bacteria-infected wounds modeled in mice, the FNDs-MNs demonstrated distinct color variations during infected phases and successfully delivered on-demand therapeutic effects, which offer promising solutions for infection control and improved wound healing.

### Atopic Dermatitis Skin Regeneration

Skin dermatitis, also known as eczema, is a common inflammatory skin condition affecting individuals across all age groups [232]. It is characterized by red, itchy, and inflamed skin, often accompanied by rashes, blisters, or dry patches. Dermatitis can manifest as either acute which lasts for a short duration or chronic which persists over a longer period. It can be present in various forms including AD, contact dermatitis, or solar dermatitis. The exact causes of dermatitis are not fully understood, but it is believed to result from a combination of genetic predisposition, environmental factors, and an overactive immune response [233]. Managing dermatitis typically involves a combination of preventive measures and treatment options including the use of topical corticosteroids, antihistamines, etc. [234]. However, they provide only temporary relief and are not considered a definitive treatment as their prolonged use can lead to undesirable side effects. Therefore, there is an urgent need to explore alternative therapeutics for the management and treatment of dermatitis [235]. Recently, it has been recognized that increased oxidative stress is closely linked to the development of AD. Consequently, mitigating oxidative stress induced by ROS in AD lesions has emerged as a potential therapeutic approach [236]. This has spurred the development of novel nano-formulations of NZ@hydrogels with enhanced efficacy and reduced toxicity [237].

**Fig. 7:**
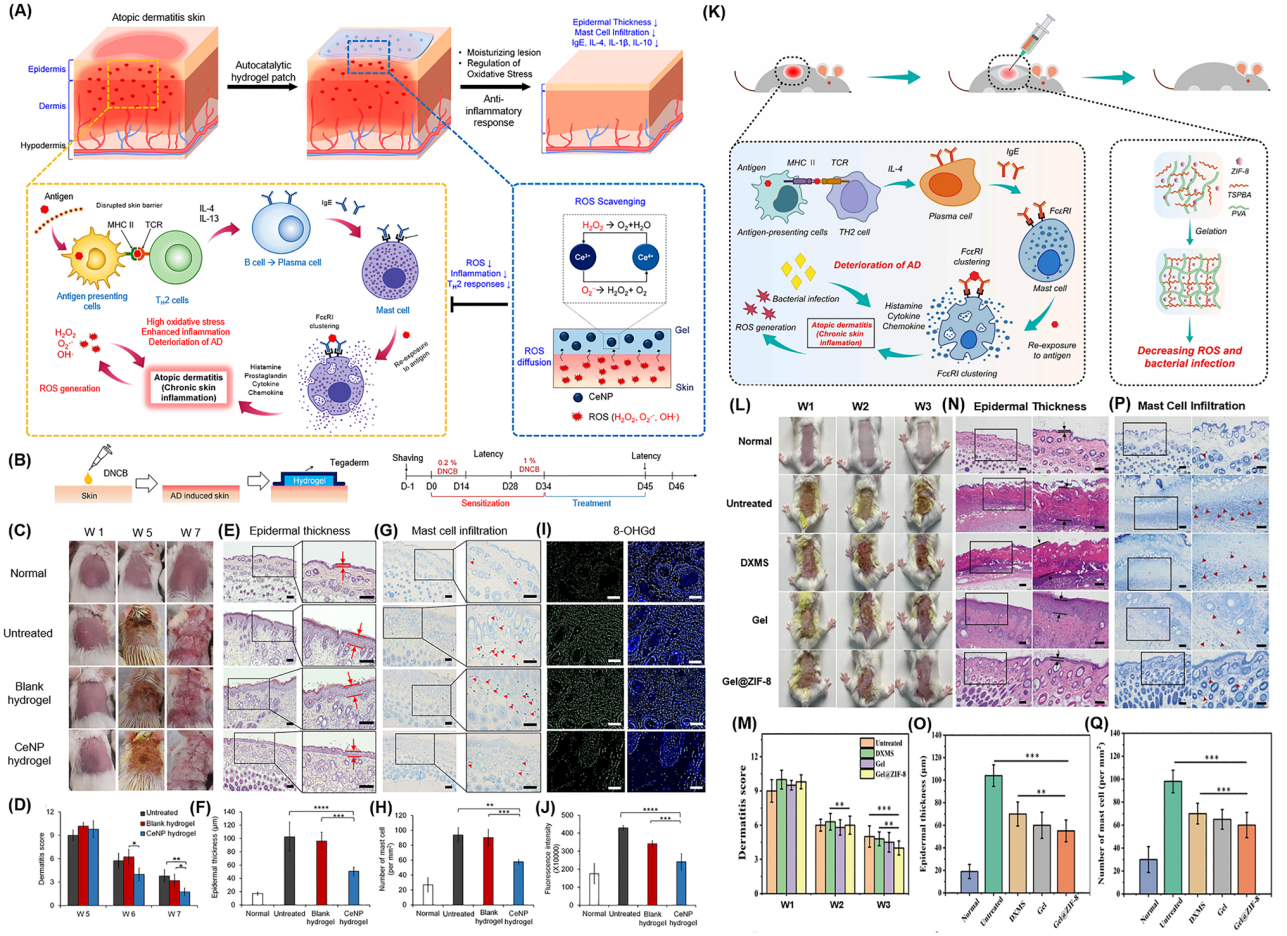
Therapeutic properties of NZ@hydrogels in alleviating AD. **a** Illustration of the design of a nCe-based hydrogel patch to treat AD. **b** Schematic of in vivo experiments and timeline for animal experiments. **c** Photographs of dorsal skin of different groups following treatment. **d** Evaluation of dermatitis score. **e** Histology of mouse skin sections stained with H&E after treatment. Scale bars, 100 μm. **f** Comparison of epidermal thickness. **g** Histology of the samples revealed infiltrated mast cells. Scale bars, 100 μm. **h** Relative quantification of infiltrated mast cells. **i** Representative immunofluorescence images of oxidative DNA damage marker: 8-OHdG. Scale bars, 100 μm. **j** Relative 8-OHdG quantification [49]. Copyright 2022, American Chemical Society. **k** An illustration highlighting the capacity of Gel@ZIF-8 to suppress oxidative stress and alleviate inflammatory responses. **l** Photographs of mice dorsal skin following treatment. **m** Evaluation of dermatitis score. **n** Comparison of epidermal thickness between groups. Scale bars, 100 μm. **o** Relative quantification of epidermal thickness. **p** Histology of the samples showing infiltrated mast cells. Scale bars, 100 μm. **q** Relative quantification of mast cells [239]. Copyright 2023, Springer Nature

A recent study by Kim et al. introduced a novel approach for treating AD using therapeutic hydrogels capable of scavenging detrimental ROS [49]. The hydrogels consist of alginate embedded with nanoceria (nCe), known for their antioxidant properties facilitated by a cyclic redox reaction between Ce^3+^ and Ce^4+^ oxidation states. The nCe-engineered alginate hydrogel was created through pH-controlled cross-linking of the alginate polymer with Ca^2+^ The composite hydrogels also demonstrated favorable mechanical properties, biocompatibility, and multienzymatic properties resembling CAT and SOD (Fig. 7a). In animal studies where the skin was directly exposed to H_2_O_2_ or DNCB, the hydrogels demonstrated therapeutic effectiveness after direct application onto AD lesions. The beneficial effects were observed through various indicators including a reduction in epidermal thickness, diminished accumulation of DNA oxidation damage products, decreased levels of Th2 cytokines and IgE, and a decrease in the number of infiltrating mast cells (Fig. 7b–j). After eleven days, the groups treated with untreated and blank hydrogels retained skin wounds, in contrast to the significantly smaller wounds observed in the NZ@hydrogel group. The dermatitis scores at week five demonstrated varying degrees of improvement with different treatments, and the NZ@hydrogel group exhibited the most substantial reduction. In terms of epidermal thickness, the untreated and blank hydrogel groups showed a sixfold and 5.6-fold increase, respectively, compared to the healthy group. In contrast, the NZ@hydrogel group displayed a two-fold decrease in epidermal thickness, surpassing that of the untreated group. Mast cell infiltration, as visualized through TB staining, was notably lower in the NZ@hydrogel group. The application of the blank and NZ@hydrogel significantly reduced elevated oxidative stress, as indicated by increased 8-OHdG levels by approximately 21 and 44%, respectively. All these findings suggest that ROS-scavenging hydrogels could be employed as therapeutic patches to treat and manage AD effectively.

Jia et al. also developed a multifunctional hydrogel dressing capable of alleviating the symptoms of AD [238]. Interestingly, in this study, the authors additionally aimed to inhibit focal adhesion kinase (FAK), which is a mechano-signaling component while simultaneously addressing oxidative stress for effective management of AD. The symptoms of AD often worsen due to bacterial infection and the resultant damage to blood vessels and endothelial cells. Therefore, an effective approach for managing AD also involves the elimination of microbes and regulation of oxidative stress on the skin surface [233]. To address this issue, a recent study led by Qiu et al. developed a hydrogel incorporating ZIF-8, referred to as Gel@ZIF-8 [239]. This hydrogel demonstrated remarkable ROS responsiveness, antibacterial effects, and high biocompatibility (Fig. 7k). Gel@ZIF-8 was formed by cross-linking PVA and ROS-responsive linker called TSPBA through a quaternization reaction forming an aryl borate polymer. The B–C bond within the structure can be broken in response to ROS resulting in the formation of phenol. This unique feature makes hydrogel ROS-responsive while maintaining favorable physicochemical properties. Furthermore, ZIF-8 in the hydrogel could release Zn^2+^ ions, disrupting bacterial integrity. The rough surface of the ZIF-8 also enhanced the contact area between the NZs and bacteria, leading to improved antibacterial effects. In an animal model of AD, the administration of Gel@ZIF-8 demonstrated therapeutic efficacy as evidenced by reduced epidermal thickness, reduced infiltration of mast cells, and reduced levels of IgE (Fig. 7l–q). After 14 days of treatment, the Gel@ZIF-8 group exhibited smaller wounds compared to other groups. The Gel@ZIF-8 group showed a two-fold reduction in epidermal thickness compared with the untreated group. Toluidine blue (TB) staining revealed the lowest mast cell infiltration in the dermis of the Gel@ZIF-8 group. Dermatitis scores indicated a similar severity at week 1, decreasing with treatment. The ZIF-8 hydrogel group exhibited the lowest dermatitis score, which was statistically significant. The evaluation of AD-related immune proteins showed decreased IgE levels in the pristine hydrogel group, while Gel@ZIF-8 continued to exhibit therapeutic effects. These findings suggest that this NZ@hydrogel design has the potential to modulate the inflammatory microenvironment and therefore presents a hopeful and promising avenue for the treatment and management of AD.

### Radiation-Induced Skin Wound Healing

RISI refer to the development of injuries on the skin because of radiation exposure [240, 241]. These wounds can occur due to various factors such as accidental exposure to radiation sources, radiation therapy for cancer, or occupational radiation exposure. Radiation exposure can disrupt the basal cell layer of the skin resulting in inflammation, redness, and the emergence of dry or moist skin peeling [242]. Furthermore, it can also lead to hair loss due to damage to the hair follicles and thereby impair the regeneration and repair mechanisms of the skin. These wounds can manifest in different forms including burns, ulcers, or chronic non-healing wounds and their severity depends on factors such as the radiation dose, duration of exposure, etc. [243]. Despite the severity of RISI, most of the treatment options are simply to alleviate symptoms to help the healing process, which typically involves pain relievers or anti-inflammatory creams.

Nanotherapeutic approaches have recently demonstrated significant efficacy in addressing RISI [244]. In a recent approach, microRNA therapy employed in the mesoporous silica (MS)-nCe composites was designed to resolve the radiation-induced ROS [245]. These nanocomposites could counteract radiation-induced ROS and HIF-1α activation that leads to enhanced healing in radiation-induced wounds. To further enhance the therapeutic efficacy, microRNA 129 (miR129) was attached to MS-nCe using iRGD-grafted PEG. Thus, formulated nano-miR129 exhibited improved stability and antibacterial properties, and the miR129 delivered to wound sites induced the formation of effective blood capillaries with low toxicity, ultimately showing excellent radio resistance in a subcutaneous RISI modeled in mice.

Radiation-induced skin conditions also include cancerous forms such as melanoma characterized by the development of malignant tumors originating from melanocytes, the cells responsible for producing melanin [246]. Prolonged or intense exposure to UV radiation is a significant contributing factor to the development of melanoma. The harmful UV rays can damage the DNA within skin cells, which leads to the uncontrolled growth of melanocytes and forms cancerous lesions, which are considered as one of the most dangerous forms of skin cancer [247, 248]. Current treatments often involve surgical excision combined with chemotherapy or radiotherapy. However, incomplete surgical resection frequently leads to recurrences and the chronic wounds left behind are challenging to heal naturally and prone to secondary injury through infection [249, 250]. Consequently, there is an unrelenting need for therapeutic biomaterials that can simultaneously target and eliminate tumors while promoting the regeneration of skin tissue to enhance therapeutic efficacy [251].

Addressing these challenges, Wu et al. proposed a therapeutic hydrogel called MCSA that is composed of manganese-imbued calcium silicate nanowires encapsulated into alginate hydrogels [252]. This hydrogel aims to achieve in situ photothermal removal of melanoma followed by skin regeneration. The MCSA hydrogel possessed tunable gelation and mechanical characteristics along with exceptional bioactivity attributed to the incorporation of calcium silicate nanowires as in situ cross-linkers and bioactive components. Manganese incorporation in the calcium silicate nanowires provided excellent photothermal effects that effectively eradicate melanoma under NIR irradiation. Furthermore, the synergistic effect of manganese significantly promoted the migration and growth of vascular endothelial cells thus facilitating angiogenesis. Consequently, these bioactive hydrogels could simultaneously perform PTT for melanoma and promote wound healing which makes them highly promising for the treatment of melanoma and tissue regeneration.

**Fig. 8:**
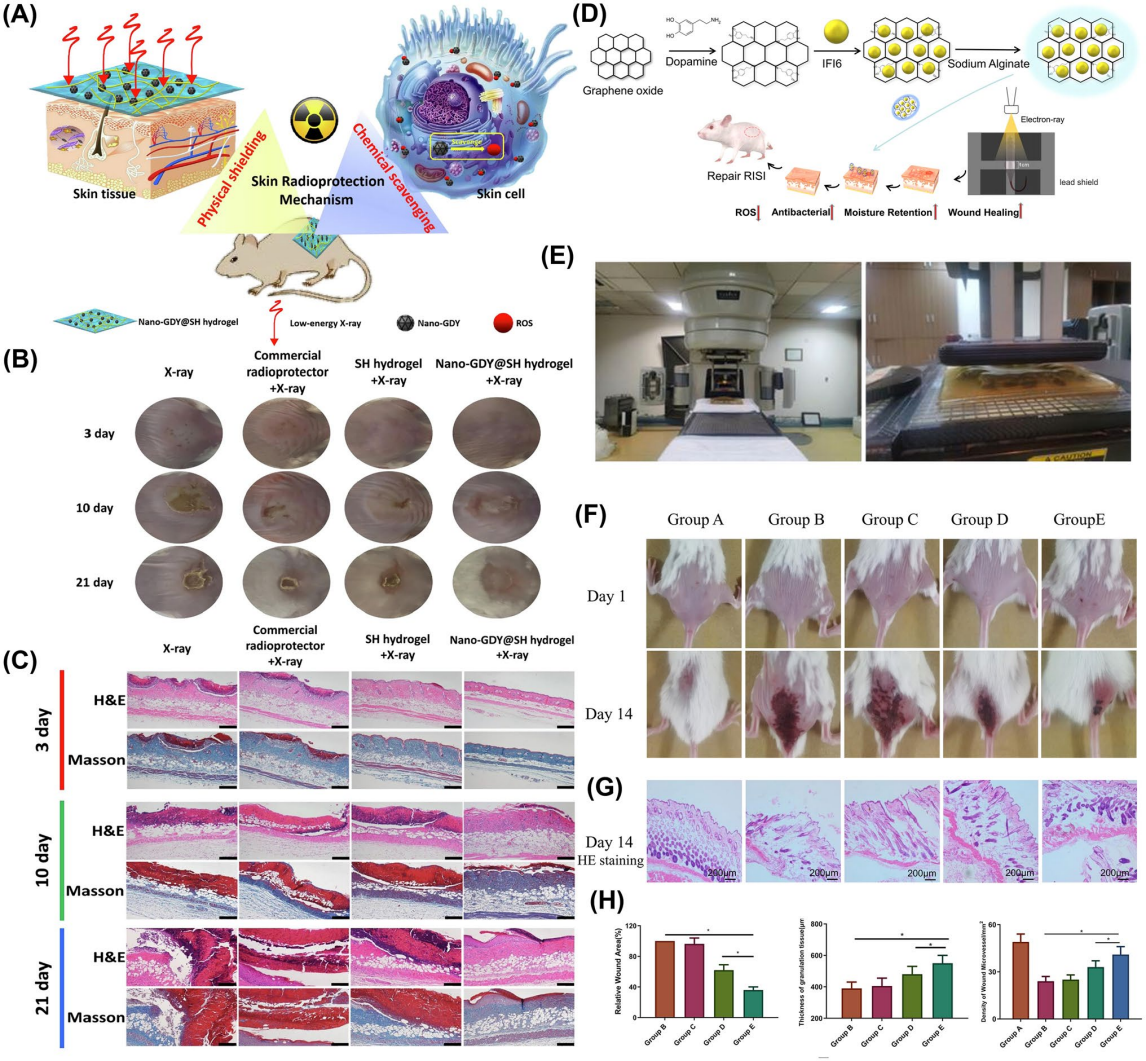
NZ@hydrogels act as radioprotectants for skin therapy. **a** Graphical representation showing the skin radioprotection capacity of the nano-GDY@SH hydrogel. **b** The photos of the skin wound changes of mice after different treatments. **c** The examination of skin tissues by H&E and Masson staining Scale bars, 100 μm [258]. Copyright 2021, Elsevier. **d** Schematic depicting the fabrication of IFI6-PDA@GO/SA hydrogel for RISI. **e** Images showing medical device used to induce the RISI model in mice. **f** Photographs of the dorsal skin of mice during the treatment period. **g** H&E staining of skin tissues on the 14th day. Scale bars, 200 μm. **h** Relative quantification of the wound area, granulation tissue thickness, and density of wound micro vessels [259]. Copyright 2022, Springer Nature

Radiation not only fails to aid tumor handling but also inflicts severe skin damage by directly striking DNA or incidentally bringing unwarranted free radicals that harm skin cells [253, 254]. Therefore, a practical approach to protect the skin from harmful radiation involves physically shielding it to minimize interaction with skin cells [255, 256]. Currently, the commercially available skin radioprotective medications primarily rely on a particular chemical repair pathway to eradicate intracellular radicals, which leads to limited therapeutic outcomes. In this context, NZ@hydrogels presents a promising solution possessing enzymatic and antioxidant capabilities. They offer a viable avenue for highly efficient radioprotective agents in situations involving radiotherapy and nuclear radiation accidents [257]. For instance, Xie et al. developed a nanosized graphdiyne-loaded sodium hyaluronate hydrogel (nano-GDY@SH hydrogel) that exhibits potent holistic free radical scavenging activity through nano-GDY and excellent low-energy X-ray attenuation ability due to the high-water content of hydrogel [258]. Test results revealed that the nano-GDY@SH hydrogel was biologically safe and effectively decreased low-energy X-ray-induced edema and skin ulcers in mice by reducing skin damage duration and promoting wound recovery (Fig. 8a–c). After 3 days of treatment, noticeable edema was observed in all groups except for the nano-GDY@SH hydrogel group, which showed only slight edema. On the 10th day, the nano-GDY@SH hydrogel group showed minimal damage with slight ulcers while other groups exhibited severe damage characterized by the largest ulcer area. By the 21st day, the non-treated group still displayed the largest ulcer area, whereas the nano-GDY@SH hydrogel group demonstrated a noticeable trend of recovery. Except for the non-treated group, all hydrogel groups demonstrated different levels of hair regeneration and the nano-GDY@SH hydrogel group displayed the most rapid regeneration. Histopathological analyses revealed that nano-GDY@SH hydrogels maintained an almost intact epidermal structure with minor damage, whereas other groups displayed serious interstitial edema and inflammatory infiltration, which were most severe in the non-treated group. Masson staining highlights the relatively orderly arrangement of collagen fibers in the nano-GDY@SH hydrogel group, exhibiting minimal scattering. In contrast, other groups showed obvious scattering with the most severe effects observed in non-treated group.

Sprayable NZ@hydrogels represent an innovative and versatile approach to skin regeneration. By harnessing their unique properties, researchers and clinicians have the potential to revolutionize RISI treatment, which facilitates more effective healing and improved patient outcomes. In a recent study, a sprayable composite hydrogel was developed composed of SA, GO, interferon-alpha inducible protein 6 (IFI6), and PDA. The IFI6-PDA@GO/SA hydrogel demonstrated the ability to enhance the proliferation and migration of skin cells thereby conferring synergistic radio resistance both in vitro and in vivo [259]. Furthermore, the portability and convenience of sprayable therapeutic hydrogel, along with its dual capacities for countering ROS and promoting skin wound healing, synergistically enabled successful intervention in a mouse model of RISI. This was evident from the ameliorated inflammation, stimulated granulation tissue formation, enhanced angiogenesis, and improved collagen deposition (Fig. 8d–h). The in vivo experiments validated the significant promotion of wound healing by IFI6-PDA@GO/SA hydrogel compared to other groups. On day 14, inflammation was observed in all groups, with NZ@hydrogel groups forming an epidermis. The total healing time analysis revealed that NZ@hydrogel exhibited the strongest effect, achieving complete healing in about 26 days, significantly surpassing other groups. Histological analysis demonstrated that IFI6-PDA@GO/SA substantially increased granulation tissue thickness and wound blood vessel density, further supporting its superior wound healing promotion compared to other groups.

## Consideration of NZ@Hydrogels with Mechanobiological Functions

Skin is constantly exposed to a variety of environmental factors including fluctuations in temperature, mechanical damage, radiation, infections, chemicals, and allergens. These factors have the potential to disrupt the natural balance of skin affecting its mechanosensitive pathways and contributing to the emergence of inflammatory skin disorders [260–262]. Inflammatory processes within the skin lead to significant changes in its cellular composition. This involves the production of pro-inflammatory cytokines by keratinocytes, fibroblasts, and immune cells as well as elevated infiltration of immune cells into the dermis and epidermis. Additionally, there is an excessive proliferation and abnormal differentiation of keratinocytes that compromises the function of the intercellular barrier and ECM remodeling by fibroblasts [263]. Many investigations have highlighted that various cell types within the skin can perceive mechanical cues [264, 265]. However, our understanding of the precise role played by mechanical signals and mechano-transduction in the development of skin inflammation remains limited.

AD or eczema serves as a prime example of a multifactorial disorder influenced by both genetic and environmental factors where mechanical signaling plays a significant role [266]. Mechano-transduction mechanisms play a crucial role in the development and advancement of AD and their disruptions can serve as triggering factors for various skin pathologies [267]. Many recent studies underscore the significance of mechano-transduction signaling molecules including integrins, focal adhesion molecules, actin cytoskeleton, and nuclear transcription factors [268–270]. For instance, focal adhesion kinase (FAK) has been considered a pivotal convergence point for different mechano-transduction pathways associated with inflammatory skin disorders [271, 272]. FAK can translocate to the nucleus and modulate the expression of genes related to inflammation such as chemokines and cytokines and the secretion of ECM molecules like collagens thereby impacting the composition of the ECM [271]. Consequently, FAK can serve as a mediator for both inflammatory and physical signals in AD [273].

**Fig. 9:**
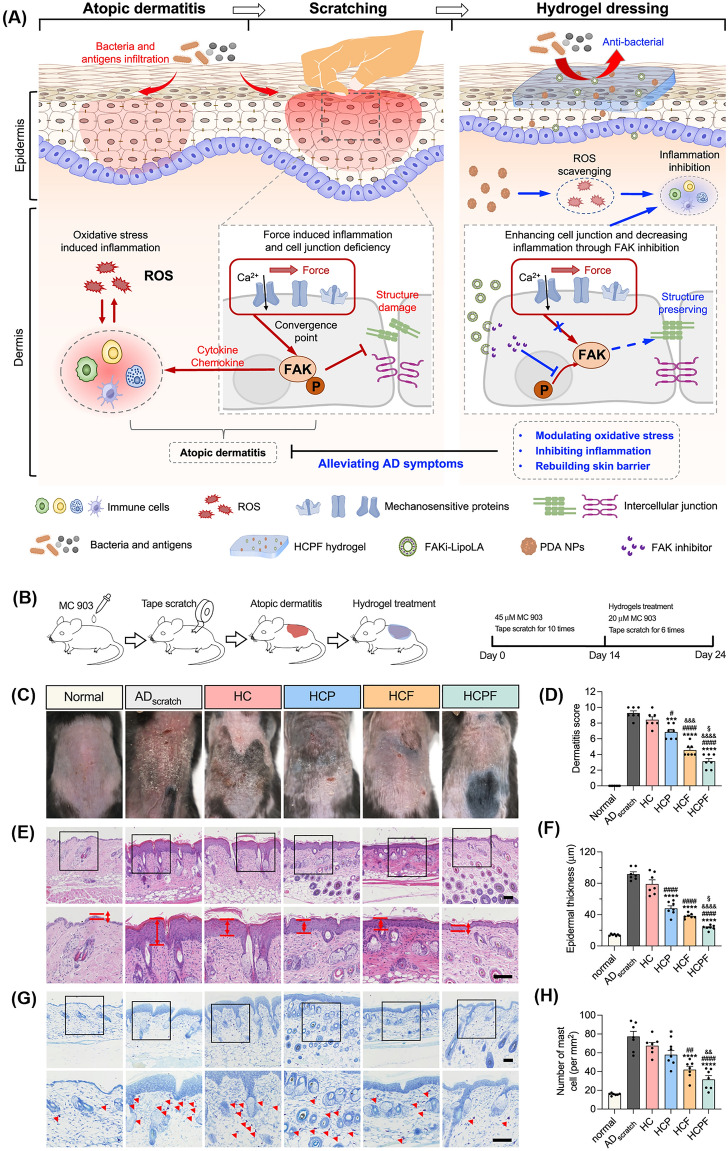
NZ@hydrogels act mechano-chemically in alleviating symptoms of AD. **a** Graphical illustration displaying inflammatory reactions of AD due to oxidative harm and the outcome of mechanical scratching on AD. It also depicts the synergistic action of NZ@hydrogels in treating AD by ROS scavenging and FAK phosphorylation inhibition. **b** Representation of in vivo experiments. **c** Photographs of the dorsal skin of mice on the 24th day. **d** Evaluation of dermatitis score. **e** H&E staining of skin sections. Scale bars, 100 μm. **F** Epidermal thickness quantified from H&E staining. **g** TB staining of the skin section. Scale bars, 100 μm. **h** Measurement of the density of mast cells for each group after treatments [238]. Copyright 2023, Springer Nature

Recently, Jia et al. developed a hydrogel dressing termed HCPF for the treatment of AD that exhibits adhesive, stretchable, and self-healing properties [238]. Dopamine-functionalized hyaluronic acid (HA-DA) and phenylboronic acid (PBA)-functionalized carboxymethyl chitosan (CMCS-PBA) served as the hydrogel backbone. PDA NPs and lauric acid-loaded liposomes (lipoLA) loaded with defactinib, a FAK inhibitor (FAKi-lipoLA) in monodisperse form were introduced individually or in combination into the hydrogel networks for synergistic outcomes. LipoLA is known for its antimicrobial activity and has been extensively utilized in treating various skin conditions. Therefore, lipoLA was selected as the carrier for delivering FAKi to target external mechanical stimulation in AD skin. The PDA NPs are known for their ROS-scavenging properties due to their abundance of reducing catechol groups (Fig. 9a). The researchers validated the ROS-scavenging efficiency of PDA NPs and the protective effect of FAKi-lipo on cells subjected to mechanical stimulation in vitro. Furthermore, using a controlled mechanical scratching mouse model of AD, they proved the mechano-chemical synergistic therapy of the hydrogel patch. A mouse model of AD with controlled mechanical stimulation was employed to assess the combined therapeutic efficacy of HCPF hydrogels. After a 10-day NZ@hydrogel treatment, both the HCF and HCPF groups exhibited a significant reduction in pFAK expression, indicating successful regulation of FAK phosphorylation facilitated by FAKi-lipoLA delivery through the hydrogel. Additionally, skin lesions significantly improved with the administration of PDA NP-loaded HCP hydrogels and FAKi-lipoLA-loaded HCF hydrogels. Notably, the synergistically treated HCPF group had the lowest dermatitis score, showing minimal dandruff and mild edema compared to other groups. Furthermore, HCPF demonstrated significant alleviation of epidermal hypertrophy and minimal mast cell infiltration. These findings collectively suggest the effectiveness of HCPF hydrogels in improving the skin pathology of AD in the presence of scratching (Fig. 9b–h). Altogether, this study validates that the integration of ROS scavenging and FAK inhibition within the NZ@hydrogel holds great promise as a synergistic treatment approach for AD. This ultimately highlights the significance of cellular mechanobiological aspects in a broad spectrum of skin wound therapies, warranting further explorations of NZ@hydrogels with mechanobiological functions as an exciting research area.

## Outlook and Challenges

As discussed, the NZ@hydrogels have proven versatile platforms for treating various skin complications through counteracting ROS that reduces inflammation, combats bacteria, and even exhibits anti-cancer properties [274]. Their increased applicability is due to the capacity to overcome limitations associated with other existing nanomaterial-based platforms. Conventional treatments often involve generic topical creams or oral medications that aim to suppress immune responses or alleviate symptoms in a non-specific manner. On the other hand, NZ@hydrogel therapies possess the ability to catalyze specific reactions involved in resolving inflammation, which leads to a more precise and efficient treatment approach [38]. Furthermore, conventional therapies often suffer from limited localization at the specific sites of inflammation due to their systemic or topical administration methods. In contrast, NZ@hydrogels offer a controlled and localized delivery of therapeutic agents. The hydrogel structure retains the NZs and gradually releases them ensuring a sustained and targeted therapy. This approach combined with the enzymatic properties significantly enhances treatment efficacy and expedites the wound healing process.

**Fig. 10:**
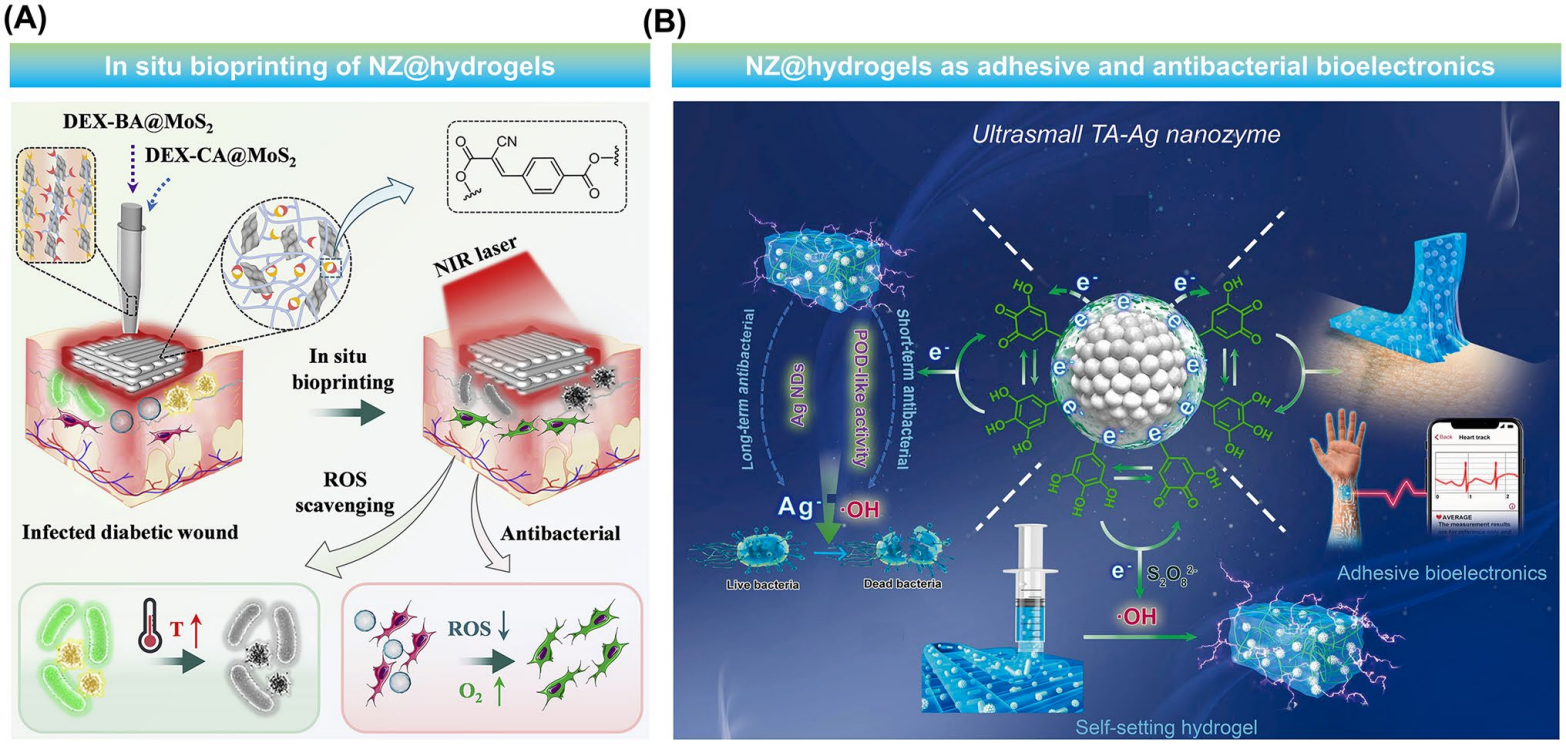
Design of advanced NZ@hydrogel platforms for skin therapeutics. **a** Making of MoS_2_-aided gelling hydrogel scaffolds through a microfluidic-assisted in situ printing approach to expedite the healing of infected chronic wounds [86]. Copyright 2023, Elsevier. **b** Ultrasmall TA-Ag NZ-catalyzed hydrogel platform is a sticky, antibacterial, and implantable bioelectrode that detects bio-signals and accelerates tissue regeneration while preventing infection [65]. Copyright 2021, Elsevier

For therapeutic hydrogels incorporating NZs, future focus should be directed toward advancing microfabrication techniques. By combining nano/micro-engineering techniques with bio-fabrication methods, it is possible to create advanced NZ@hydrogels with customized properties specifically for engineering skin tissue equivalents [275–278]. These advanced technologies provide precise control over the composition, structure, and functionality of NZ@hydrogels that result in enhanced biocompatibility, interactions between cells and materials, and improved therapeutic outcomes. Techniques such as 3D bioprinting can be employed to create hydrogels that closely imitate the intrinsic ECM of the skin [48, 279–281]. For instance, in a recent study, a microfluidic-assisted in situ printing technique was used to develop MoS_2_-accelerated gelling hydrogel scaffolds to speed up the healing of infected wounds [86]. By incorporating MoS_2_ NSs, which are known for their exceptional photothermal properties and ROS-scavenging ability, the resulting hydrogel scaffolds not only alleviated oxidative stress but also displayed remarkable antibacterial efficacy (Fig. 10a). Furthermore, the study provided evidence that MoS_2_ hydrogel scaffolds can be directly printed onto chronic diabetic wounds showing a perfect fit and strong adherence to the irregular shapes of the wounds. Concurrently, the printed hydrogel constructs offer mechanical support and a biomimetic setting for various cellular processes.

In another study, 3D printing technology was used to create a nCe-based MOF hydrogel for personalized wound dressings [67]. This hydrogel stands out as a nCe-based MOF that is seamlessly integrated into the hydrogel network thereby streamlining the MOF hydrogel printing process. The hydrogel exhibited remarkable enzymatic activity toward various free radicals and enabled indirect monitoring of glucose levels surrounding the wound. Furthermore, the hydrogel exhibited excellent antibacterial properties and biocompatibility. The combined anti-inflammatory and hyperglycemic capacity of the nCe-based MOF hydrogel synergistically contributed to enhanced healing of diabetic wounds. Microfabrication techniques such as photolithography or soft lithography can also be employed to generate microscale patterns or gradients of NZs within the hydrogel matrix [282]. By controlling the spatial distribution of NZs, it is possible to create regions within a hydrogel with varying enzymatic activities or functions. This enables the creation of intricate enzymatic microenvironments that can influence specific cellular behaviors or guide skin tissue regeneration [32, 283].

An additional decisive feature of the NZ@hydrogel platforms is their capacity for intelligence, which makes them able to detect and react to specific stimuli [100]. This responsiveness can be initiated by diverse stimuli such as pH, temperature, light, or specific biomolecular signals that allow for the precise regulation of various actions. Stimuli-responsive NZ@hydrogels offer a dynamic, bioactive, and customizable environment that has great potential for facilitating scarless healing and addressing several skin conditions. Stimuli-responsive NZ@hydrogels could also be used for real-time monitoring of wound closure, which enables noninvasive assessment of the wound healing process. Stimuli-responsive NZ@hydrogels can also be designed to be minimally invasive for easier applications in clinical settings [284]. For example, a recent study by Shan et al. developed intelligent MN patches with both therapeutic and sensing capabilities to efficiently detect and eradicate bacteria in skin wounds [231]. This intelligent MN exhibited POD-mimic activity and underwent color changes depending on the pH and H_2_O_2_ concentration, which provides timely information on the wound healing progress and infection status as demonstrated in bacteria-infected wounds.

Likewise, the ultrasmall TA-Ag NZ-catalyzed hydrogel platform developed by Jia also could function as an intelligent NZ@hydrogel that effectively acts as a sticky, antibacterial, and implantable bioelectrode that detects biosignals and accelerates tissue regeneration while preventing infection [65] (Fig. 10b).

Another promising area of research involves the integration of NZ@hydrogels with gene therapy or stem cell-based approaches. The inherent catalytic activity of the NZ@hydrogels plays a crucial role in modulating the cellular microenvironment by oxidative stress [285]. Moreover, therapeutic stem cells have immunomodulatory properties that regulate the immune response in the wound microenvironment, thus supporting the survival of stem cells and enhancing their regenerative ability [286]. This approach combines the regenerative potential of stem cells with the unique attributes of NZs, leading to improved effectiveness in tissue engineering strategies, ultimately resulting in enhanced tissue regeneration and more effective treatment of inflammatory skin diseases.

The future of NZ-based platforms is also widening with data-driven intelligent design and the potential use of machine-learning (ML) technology [154]. The ML, with its computational algorithms, aims to infer mathematical models from existing data and thereby offers an efficient tool for accelerating the development of desired materials. Traditionally, the synthesis of NZs for the development of composites with specific characteristics relies on inefficient and resource-intensive techniques. However, ML has provided a means to uncover the intricate connections between NZ physicochemical features and enzyme-like functionalities. Despite these promises, the platforms also face several challenges such as the absence of standardized evaluation methods, complexity-accuracy tradeoffs in ML models, and challenges stemming from high dimensionality and limited sample data. Overcoming these will be crucial for fully harnessing the potential of data-driven intelligent design and ML in advancing NZ@hydrogel research for skin therapies.

Despite their potential applications in skin regeneration, NZ@hydrogels have inherent limitations that need to be considered. Biocompatibility and potential toxicity are the main concerns as certain NZs may trigger adverse reactions or immune responses within biological systems, which demands rigorous safety evaluations [287]. Moreover, the long-term effects of NZ@hydrogels in the body remain incompletely understood, demanding extensive research into potential chronic implications including its accumulation and biodegradation [288]. Cost-efficiency represents another major concern, as the manufacturing and quality assurance of NZ-based systems frequently experience large expenses that limit their accessibility. Similarly, consistency in manufacturing processes and quality control are also essential to ensure the reproducibility of NZ@hydrogels [289]. Additionally, achieving specificity and selectivity like natural enzymes can be challenging and issues such as stability, optimization, and efficient delivery to target sites require careful attention [290]. Additionally, concerns regarding the in vivo efficiency of these materials further underline the need for a thorough investigation and responsible use of NZ@hydrogels.

Even though NZ@hydrogels represent a promising frontier in various clinically relevant tissue conditions, several challenges need to be addressed to harness their full potential and ensure their safe and effective use in clinical practice [22, 26, 291]. First and foremost, achieving long-term stability is of great concern as NZs and hydrogels can degrade over time which compromises their catalytic activity and functionality under physiological conditions [292]. The low selectivity of these platforms toward the target raises concerns regarding their potential toxicity and off-target effects [293]. An alternative approach to tackle the limitations of NZ@hydrogels and reduce their toxicity is through surface modification of NZs [294]. Therefore, careful consideration and selection of appropriate ligands are vital steps in improving the biosafety of NZs. Enhancing the catalytic efficiency of most NZs is crucial in achieving controllable enzyme-like activity. There is a strong need for high-defect site-specific enzymatic reactions to ensure biocompatibility and therapeutic specificity. Introducing single-atom NZs is an appealing strategy due to their well-dispersed catalytic active sites and efficient atomic utilization [295, 296]. Furthermore, a limitation of many enzyme-based platforms is their optimal catalytic activity is restricted to acidic pH, which is not compatible with physiological and biological conditions. This constraint hinders their effectiveness and practical application in real-life scenarios.

Although preclinical studies using established animal models have demonstrated promising treatment outcomes with NZ@hydrogels, our understanding of the biological mechanisms governing their interactions in vivo remains limited. Therefore, rigorous safety and efficacy evaluations of these biomaterial platforms are necessary before considering their administration in patients [293]. Furthermore, it is essential to develop more practical diagnostic methods that allow real-time monitoring of ROS levels in pathological areas of the human body [297]. Such advancements would enable the precise determination of optimal doses and administration routes for NZ@hydrogels that ultimately maximize their clinical benefits. Additionally, it is important to simplify the design of NZ@hydrogels to ensure translational feasibility rather than pursuing complex structures that may introduce potential biosafety concerns. In conclusion, NZ@hydrogels represent a rapidly evolving and promising field capable of revolutionizing the treatment of inflammation-related disorders and skin regeneration. Through ongoing research, innovation, and collaboration among researchers, biomedical engineers, and clinicians, these advanced platforms can profoundly impact the future of healthcare, providing novel therapies and enhancing the quality of life for patients worldwide.
